# Asymmetry of the Budding Yeast Tem1 GTPase at Spindle Poles Is Required for Spindle Positioning But Not for Mitotic Exit

**DOI:** 10.1371/journal.pgen.1004938

**Published:** 2015-02-06

**Authors:** Ilaria Scarfone, Marianna Venturetti, Manuel Hotz, Jette Lengefeld, Yves Barral, Simonetta Piatti

**Affiliations:** 1 Centre de Recherche en Biochimie Macromoléculaire, Montpellier, France; 2 Dipartimento di Biotecnologie e Bioscienze Università degli Studi di Milano-Bicocca, Milano, Italy; 3 Institute of Biochemistry, ETH Zurich, Zurich, Switzerland; Stowers Institute for Medical Research, UNITED STATES

## Abstract

The asymmetrically dividing yeast *S. cerevisiae* assembles a bipolar spindle well after establishing the future site of cell division (i.e., the bud neck) and the division axis (i.e., the mother-bud axis). A surveillance mechanism called spindle position checkpoint (SPOC) delays mitotic exit and cytokinesis until the spindle is properly positioned relative to the mother-bud axis, thereby ensuring the correct ploidy of the progeny. SPOC relies on the heterodimeric GTPase-activating protein Bub2/Bfa1 that inhibits the small GTPase Tem1, in turn essential for activating the mitotic exit network (MEN) kinase cascade and cytokinesis. The Bub2/Bfa1 GAP and the Tem1 GTPase form a complex at spindle poles that undergoes a remarkable asymmetry during mitosis when the spindle is properly positioned, with the complex accumulating on the bud-directed old spindle pole. In contrast, the complex remains symmetrically localized on both poles of misaligned spindles. The mechanism driving asymmetry of Bub2/Bfa1/Tem1 in mitosis is unclear. Furthermore, whether asymmetry is involved in timely mitotic exit is controversial. We investigated the mechanism by which the GAP Bub2/Bfa1 controls GTP hydrolysis on Tem1 and generated a series of mutants leading to constitutive Tem1 activation. These mutants are SPOC-defective and invariably lead to symmetrical localization of Bub2/Bfa1/Tem1 at spindle poles, indicating that GTP hydrolysis is essential for asymmetry. Constitutive tethering of Bub2 or Bfa1 to both spindle poles impairs SPOC response but does not impair mitotic exit. Rather, it facilitates mitotic exit of MEN mutants, likely by increasing the residence time of Tem1 at spindle poles where it gets active. Surprisingly, all mutant or chimeric proteins leading to symmetrical localization of Bub2/Bfa1/Tem1 lead to increased symmetry at spindle poles of the Kar9 protein that mediates spindle positioning and cause spindle misalignment. Thus, asymmetry of the Bub2/Bfa1/Tem1 complex is crucial to control Kar9 distribution and spindle positioning during mitosis.

## Author Summary

In asymmetrically dividing cells, proper positioning of the mitotic spindle relative to polarity determinants is crucial to ensure the unequal fate of daughter cells. In stem cells, derangement of the mechanisms controlling asymmetric cell division, including spindle positioning, affects the developmental fate of daughter cells and can promote tumourigenesis. The budding yeast *Saccharomyces cerevisiae* is an outstanding model system to study spindle positioning and its links with cell cycle progression. Indeed, budding yeast has redundant mechanisms driving spindle positioning and a “spindle position checkpoint” (SPOC) that delays cell division whenever the spindle is not properly aligned. The target of the SPOC is the small GTPase Tem1 that controls both spindle positioning and mitotic exit and whose activity can be inhibited by the GTPase-activating protein Bub2/Bfa1. Tem1, Bub2 and Bfa1 form a complex at spindle poles that becomes asymmetric and accumulates on one spindle pole when the spindle is properly aligned, while it remains symmetric in case of spindle mispositioning. Through expression of several mutant or chimeric proteins leading to symmetric distribution of the Bub2/Bfa1/Tem1 complex, we establish that asymmetry of these proteins does not drive mitotic exit but rather it contributes to spindle alignment.

## Introduction

Asymmetric cell division generates two daughter cells genetically identical but that differ in fate and/or in size and cytoplasmic material. During asymmetric cell division, polarity factors are first concentrated to specific locations to define the poles of cell division. Afterwards the spindle orients according to these polarity cues to segregate one set of chromosomes towards a given polarity determinant and the other away from it, thereby generating two unequal daughter cells (reviewed in [1–3]). Correct spindle positioning is therefore critical to preserve the right lineage of asymmetrically dividing cells. Accordingly, spindle mispositioning in asymmetrically dividing stem cells, which normally generate one daughter stem cell with self-renewal potential and one cell destined to differentiation, steers tumourigenesis by increasing the pool of undifferentiated stem cells [4, 5]. Surveillance mechanisms, or checkpoints, must therefore respond to spindle positioning errors and delay cell cycle progression until the mitotic spindle is properly oriented with respect to the cell polarity axis [6, 7].

The budding yeast *Saccharomyces cerevisiae* is a widely recognized model system to study asymmetric cell division. Spindle positioning in budding yeast requires either one of two redundant pathways, one that depends on the APC (Adenomatous Polyposis Coli)-related protein Kar9, and the other on dynein (reviewed in [8]). Spindle positioning errors are monitored by a surveillance mechanism, referred to as spindle position checkpoint (SPOC), that delays mitotic exit and cytokinesis to provide the time for proper spindle realignment (reviewed in [6, 9]). The target of the SPOC is a small GTPase called Tem1, which acts as molecular switch for the activation of a kinase cascade related to the Hippo pathway and named Mitotic Exit Network (MEN). In the fission yeast *S. pombe* a kinase cascade similar to MEN and referred to as Septation Initiation Network (SIN) triggers cytokinesis [10]. The MEN effector of Tem1 is the kinase Cdc15, which in turn promotes the activation of the downstream Mob1/Dbf2 kinase complex that ultimately leads to activation of the Cdc14 phosphatase [11]. Cdc14 is the main phosphatase that in budding yeast counteracts the activity of cyclin-dependent kinases (CDKs), and it is essential for mitotic exit and cytokinesis by dephosphorylating CDK substrates, as well as by triggering inactivation of mitotic CDKs [12]. Cdc14 is sequestered in the nucleolus in an inactive form throughout most of the cell cycle, until it is released and activated. Although the MEN is necessary for the full release of Cdc14 into the cytoplasm to promote mitotic exit [13, 14], another pathway called FEAR (Cdc Fourteen Early Anaphase Release) causes a partial release of Cdc14 from the nucleolus into the nucleus at the metaphase-to-anaphase transition [15]. The FEAR pathway involves the polo kinase Cdc5, the redundant Spo12 and Bns1 proteins, and the separase Esp1, which by inhibiting the phosphatase PP2A^Cdc55^ allows the dissociation of Cdc14 from its nucleolar inhibitor Net1 [15, 16]. The FEAR-mediated activation of Cdc14 in anaphase is thought to regulate spindle dynamics and to contribute to timely activation of the MEN (reviewed in [17]).

Recent data have shown that MEN is not only important for triggering mitotic exit in telophase, but also has an earlier function in metaphase to promote correct spindle positioning along the polarity axis [18]. In most MEN mutants, except for *cdc14*, spindles are indeed misoriented relative to the cell division plane. Notably, the Mob1/Dbf2 kinase was found to phosphorylate the spindle positioning Kar9 protein, thereby favouring its concentration on astral microtubules emanating from only one of the two spindle poles [18]. Asymmetric distribution of Kar9 at spindle poles in metaphase is in turn crucial for proper spindle positioning because it targets the Kar9-decorated aster to the bud, due to Kar9 interaction with the type V myosin Myo2 [19].

The yeast centrosomes, named spindle pole bodies (SPBs), play an important role in the regulation of mitotic exit, as they act as a scaffold for MEN components, such as Tem1 and its downstream kinases (reviewed in [20]). The constitutive SPB component Nud1 recruits MEN proteins to SPBs and is essential for mitotic exit [21], suggesting that binding of one or several MEN factors to SPBs is required for mitotic exit. Consistently, Tem1 association to SPBs is critical for MEN activation [22].

Like all GTPases, Tem1 is active when bound to GTP and inactive in its GDP-bound form. The common element of the GTPase superfamily is the 160–180 residue G domain involved in nucleotide binding [23]. Within the G domain two flexible “switch regions”, referred to as Switch I and II undergo the most dramatic structural rearrangement upon GTP hydrolysis and therefore define the major conformational changes conferred by GTP versus GDP binding [24]. On the basis of sequence alignment with human Ras, Switch I and II in Tem1 correspond to residues 50–55 and 77–84, respectively.

Upon spindle misalignment the two-component GTPase-activating protein (GAP) Bub2/Bfa1 inactivates Tem1 by stimulating GTP hydrolysis [25, 26]. GTPase-activating proteins accelerate GTP hydrolysis, promoting the GDP-bound inactive form of GTPases [27, 28]. The GAP activity of the Bub2/Bfa1 complex resides on Bub2, which carries a TBC domain (Tre-2, Bub2 and Cdc16; [29]), whereas Bfa1 mediates Bub2 interaction with Tem1 and prevents Tem1 dissociation from guanine nucleotides, thereby acting as guanine dissociation inhibitor (GDI) [25, 26, 30, 31].

Often, the release of GDP from GTPases is a slow and thermodynamically unfavourable reaction. This is why GTPase activation requires in most cases the intervention of nucleotide exchange factors (GEFs) that catalyse the release of GDP, promoting its replacement by GTP [27]. The identity of the GEF(s) for Tem1, if any, remains elusive. The early proposal based on genetic data that the Lte1 protein might be the GEF for Tem1 has not been confirmed by biochemical assays [30]. Therefore, if inactivation of a GEF for Tem1 could play any role in the SPOC, besides Tem1 inhibition by the GAP Bub2/Bfa1, remains to be established.

The Kin4 protein kinase is a key component of the SPOC (reviewed in [6]). During spindle misalignment it phosphorylates Bfa1, thereby preventing the inhibitory phosphorylation of the GAP Bub2/Bfa1 by the polo kinase [32]. During the unperturbed cell cycle Kin4 is strategically restricted to the mother cell compartment, where it is thought to sense the anomalous persistence of both SPBs in anaphase. In addition, it is present on both SPBs of misaligned spindles [32–35].

Localization of MEN and SPOC proteins to the SPBs changes during the cell cycle, in that some proteins (like Cdc15, Mob1 and Dbf2) are loaded on both SPBs at the anaphase onset, whereas other proteins (like Tem1, Bfa1 and Bub2) are present on both SPBs already in metaphase and their localization becomes much more asymmetric in anaphase, when they preferentially accumulate on the old, bud-directed SPB [36–44]. Moreover, the position of the spindle seems to play a role in controlling the asymmetric localization of Tem1, Bub2 and Bfa1 in anaphase. Indeed, these proteins localize less strongly but more symmetrically on the two SPBs when a misoriented spindle elongates within the mother cell and the SPOC turns on [36, 38, 40]. Whether the asymmetric localization of Tem1 or its GAP is important for triggering MEN signalling remains to be elucidated. Remarkably, the SIN counterparts of several MEN components also localize asymmetrically on SPBs during anaphase, with the homologs of the GAP components Bub2 (Cdc16) and Bfa1 (Byr4) occupying one SPB, while the GTP-bound form of the GTPase Spg1 and its effector kinase Cdc7 occupy the other [45, 46]. Thus, in spite of the different modes of cell division in the two yeasts (asymmetric in *S. cerevisiae* and symmetric in *S. pombe*) MEN and SIN signalling is asymmetric in both. SIN asymmetry has been proposed to be crucial for timely cytokinesis, as *cdc16* and *byr4* mutants where Spg1 and Cdc7 are symmetric in anaphase undergo multiple rounds of septation [46, 47].

In *S. cerevisiae*, Kin4 was found to increase the turnover of the Bub2/Bfa1 at SPBs. However, Kin4 does not promote asymmetric localization of Bub2/Bfa1 on properly oriented spindles [36]. Chimeric proteins obtained by fusing Bub2 or Bfa1 to the structural SPB component Cnm67 cause unscheduled mitotic exit in the presence of mispositioned spindles, which led to the proposal that high turnover of Bub2/Bfa1 might be important to inhibit Tem1 in the cytoplasm during SPOC activation [36]. Conversely, a modified version of Bub2 carrying 9 myc epitopes at the C terminus localizes the GAP and Tem1 rather symmetrically at SPBs and prevents mitotic exit in some sensitized MEN mutant backgrounds [25].

In order to shed light onto the relationship between Bub2/Bfa1 symmetry and SPOC response, we report the characterization of a series of mutants altering either the Bub2/Bfa1 subunits or Tem1 and causing symmetric localization of Tem1 and its GAP during properly oriented anaphase. Remarkably, these mutant proteins as a whole tend to activate, rather than inhibit, the MEN. In addition, they lead to more symmetric distribution of Kar9 on spindle poles and to spindle positioning defects, indicating that a delicate balance between MEN activation and inactivation is required for proper spindle alignment.

## Results

### Bub2 GAP activity involves a ‘dual finger’ mechanism and promotes Bub2/Bfa1 disappearance from the mother SPB

**Fig 1:**
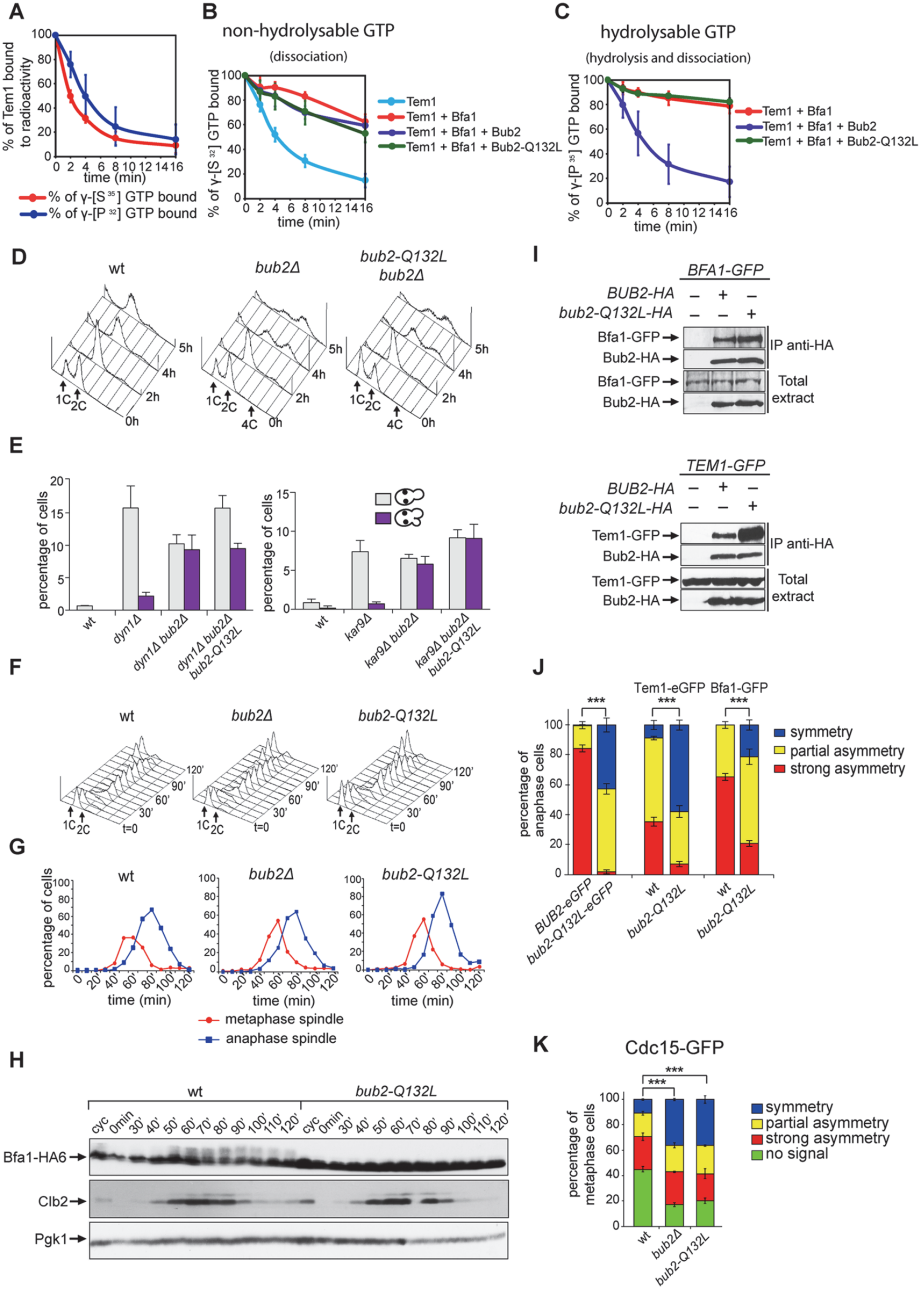
Bub2 GAP activity involves a ‘dual finger’ mechanism and promotes Bub2/Bfa1 clearance from the mother SPB. A-C: Bacterially purified GST-Bub2 or GST-Bub2-Q132L, MBP-Bfa1 and 6xHis-Tem1 proteins were used to measure the kinetics of hydrolysis+dissociation (γ[^32^P]GTP) or dissociation only (γ[^35^S]GTP) using a filter binding assay (see Materials and Methods). Graphs show average values and standard deviations from three independent experiments. D: Exponentially growing cultures of the indicated strains were shifted to nocodazole containing medium at t = 0. Cell samples were withdrawn at the indicated time for FACS analysis of DNA contents. E: The percentage of cells with binucleate cell bodies accompanied or not by a checkpoint defect (indicated by re-budding in the absence of proper chromosome segregation) was scored in cycling cultures of the indicated strains shifted either to 14°C for 16h (left graph) or to 37°C for 3h (right graph). F-G: Exponentially growing cells with the indicated genotypes were arrested in G1 by α-factor and released into fresh medium at time 0. At 70’ after release α-factor was re-added to prevent cells from entering a second cell cycle. Cell samples were collected for FACS analysis of DNA contents (F) and for tubulin staining by indirect immunofluorescence (G). H: Cells were treated as in (F-G). TCA extracts were prepared from cell samples at the indicated time points to monitor kinetics of Bfa1-HA6 phosphorylation and Clb2 accumulation and degradation by western blot analysis. Pgk1 was used as loading control. I: Protein extracts from cells expressing the indicated tagged proteins were used for immunoprecipitation with an anti-HA affinity resin. Western blot analysis was then performed with anti-GFP and anti-HA antibodies. The input represents 1/25^th^ of the total extract used for each IP. J-K: Localization of eGFP- tagged Bub2/Bub2-Q132L, Tem1, Bfa1 (J) and Cdc15-GFP (K) was analysed by fluorescence microscopy after formaldehyde fixation.

The catalytic mechanism of GTPase-activating proteins (GAPs) requires an ‘arginine-finger’, where the lateral chain of a conserved arginine (R85 for Bub2, [25, 29]), interacts with the nucleotide-binding site of a G protein, thus stimulating hydrolysis of the γ−phosphate. A new catalytic mechanism, called “dual finger”, was proposed for the family of GAPs with TBC (Tre-2, Bub2 and Cdc16) domain. According to the dual finger mechanism a conserved glutamine residue contributes to stimulate GTP hydrolysis together with the canonical catalytic arginine [48]. To investigate if Bub2 acts indeed via a dual finger mechanism, we generated a mutant Bub2 variant, Bub2-Q132L, where we replaced by leucine the conserved glutamine at position 132 that identifies the glutamine finger on the basis of sequence alignment [48]. Bacterially purified His-tagged Tem1, Maltose Binding Protein (MBP)-tagged Bfa1 and glutathione-S transferase (GST)-tagged Bub2 or Bub2-Q132L proteins were used in *in vitro* GTPase assays, as previously described [25, 26]. The rate of GTP hydrolysis and dissociation was measured using Tem1 bound to γ[^32^P]-GTP, whereas the rate of GTP dissociation alone was measured using Tem1 bound to the non-hydrolysable GTP analogue γ[^35^S]-GTP (Fig. 1A). As shown in Fig. 1A, the kinetics of radioactivity loss from wild type Tem1 loaded with either γ[^32^P]-GTP or γ[^35^S]-GTP were very similar, suggesting that Tem1 on its own mostly dissociates GTP without hydrolysing it. The presence of Bfa1 stabilized Tem1 in the GTP-bound form (Fig. 1B), whereas Bub2 stimulated Tem1 GTPase activity in the presence of Bfa1 (Fig. 1C), but not GTP dissociation (Fig. 1B). We then compared the GAP activity of purified GST-Bub2 and GST-Bub2-Q132L. Interestingly, Bub2-Q132L did not display any GAP activity towards GTP-bound Tem1 (Fig. 1C), behaving as the GAP–dead mutant Bub2-R85A previously characterized [25]. Furthermore, it did not stimulate GTP dissociation, exactly like wild type Bub2 (Fig. 1B).

*In vivo*, the Q132L substitution completely abolished the checkpoint function of Bub2. Indeed, similar to *bub2Δ* cells, *bub2-Q132L* cells escaped the mitotic arrest upon nocodazole treatment, as indicated by their ability to re-replicate their chromosomes (Fig. 1D). Checkpoint response to spindle misalignment was also impaired in *bub2-Q132L* cells. Indeed, when spindle mispositioning was induced by *DYN1* or *KAR9* deletion [49, 50] *bub2-Q132L* cells did not arrest in mitosis as large budded cells but re-budded, similar to *bub2Δ* cells (Fig. 1E). Thus, consistent with the proposed model [48], Bub2 GAP activity, and thereby its role in the SPOC, relies on a dual finger mechanism involving two catalytic residues, R85 and Q132.

The *bub2-Q132L* allele did not accelerate mitotic exit during the unperturbed cell cycle. Indeed, synchronized *bub2-Q132L* cells could divide and disassemble bipolar spindles with wild type kinetics (Fig. 1F–G). Furthermore, kinetics of degradation of the main mitotic cyclin Clb2 were very similar in wild type and *bub2-Q132L* cells (Fig. 1H). Interestingly, we found that cell cycle-dependent phosphorylation of Bfa1, which promotes mitotic exit [51], was abolished in *bub2-Q132L* cells, in agreement with the recent proposal that it requires Bub2 activity [52].

We then asked if Bub2-Q132L could still interact efficiently with Bfa1 and Tem1. Immunoprecipitations of Bub2 or Bub2-Q132L tagged with three HA epitopes showed that both proteins pulled down roughly the same amounts of GFP-tagged Bfa1. In contrast, Bub2-Q132L precipitated a higher amount of GFP-tagged Tem1 than wild type Bub2 (Fig. 1I), suggesting that abolishing the GAP catalytic activity of the Bub2/Bfa1 complex stabilizes the interaction between Tem1 and its GAP.

Because lack of Bub2 GAP activity through the *bub2-R85A* allele leads to increased symmetric localization of Bub2 to SPBs in anaphase [25], we analyzed the subcellular distribution of eGFP-tagged Bub2-Q132L. In contrast to wild type Bub2, which was almost exclusively present on the bud-directed SPB in 84% of anaphase cells, Bub2-Q132L-HA3 was found on both SPBs in 97% of cells in anaphase. In addition, Bfa1 and Tem1 were also more symmetrically localized on the SPBs of *bub2-Q132L* cells than they were in wild type cells during the same cell cycle stage (Fig. 1J). We therefore conclude that, consistent with previous data, interfering with Bub2 GAP activity affects the asymmetry of the Tem1/Bub2/Bfa1 complex on anaphase spindle poles.

Finally, we analysed the localization of the Tem1 effector kinase Cdc15 in *bub2-Q132L* cells. We found GFP-tagged Cdc15 on the SPBs of metaphase spindles in 55% of the cells. Deletion of *BUB2* or its replacement with the *bub2-Q132L* allele increased both the total percentage of cells with Cdc15 at SPBs (83% and 80%, respectively, Fig. 1K) and the percentage of cells with symmetrically localized Cdc15 in metaphase (36% in *bub2Δ* and *bub2-Q132L* cells versus 11% of wild type cells). Thus, lack of Bub2 GAP activity leads to more efficient recruitment of Cdc15 at spindle poles.

### Constitutive targeting of Bfa1 to SPBs facilitates mitotic exit by recruiting Tem1 to SPBs

**Fig 2:**
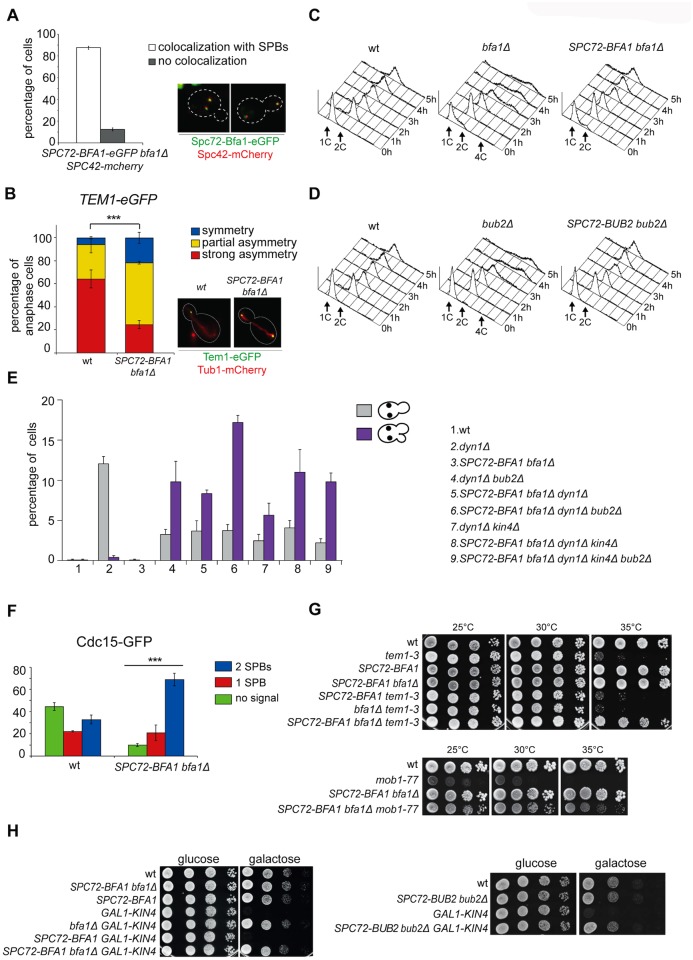
Constitutive targeting of Bfa1 to SPBs facilitates mitotic exit by recruiting Tem1 to SPBs. A-B: Cycling cells co-expressing Spc72-Bfa1-eGFPand Spc42-mCherry to mark the SPB (upper panel) or co-expressing Tem1-eGFP and Tub1-GFP (to mark microtubules, lower panel) were analysed to study the distribution of Spc72-Bfa1-eGFP (A) and Tem1-eGFP (B) at SPBs in *SPC72-BFA1 bfa1Δ* cells. C-D: Cycling cells with the indicated genotypes were shifted into nocodazole containing medium (t = 0). Cell samples were withdrawn at the indicated times for FACS analysis of DNA contents. E: The percentage of cells with binucleate cell bodies accompanied or not by a SPOC defect was scored after propidium iodide staining of cycling cultures of cells with the indicated genotypes after shift to 14°C for 16h. The histograms on the right side represent the DNA contents of the same cells as measured by FACS analysis. F: Percentage of metaphase cells with Cdc15-GFP at 0, 1 or 2 SPBs was scored in the indicated strains after formaldehyde fixation. Metaphases were identified by means of the Tub1-mCherry co-expressed marker. G: Serial dilutions of stationary phase cultures of the indicated strains were spotted on YPD and incubated at the indicated temperature. H: Serial dilutions of stationary phase cultures of the indicated strains were spotted on YP medium containing either glucose or galactose and incubated at 25°C for 48h.

Since spindle misalignment leads to persistent residence of Bub2/Bfa1 on both SPBs [40], we and others proposed that symmetric distribution of the GAP complex might lead to inhibition of Tem1 [25, 40]. This idea was further supported by our previous finding that a myc-tagged variant of Bub2 (Bub2-myc9) localizing mostly symmetrically on SPBs was lethal and prevented mitotic exit in sensitized backgrounds [25]. On the other hand, the Bub2/Bfa1 complex is required throughout most of the cell cycle for Tem1 association with SPB, which in turn triggers its activation [22, 40]. To assess the importance of Bub2/Bfa1 asymmetry at SPBs, we tethered Bfa1 or Bub2 to both SPBs by fusing them to the structural SPB component Spc72. We confirmed that in about 90% of the cells the Spc72-Bfa1 chimeric protein localised constitutively to the SPBs throughout the cell cycle (Fig. 2A) and was able to recruit Tem1 to both SPBs in 74% of anaphase cells, as opposed to 35% of wild type cells (Fig. 2B).

Both Spc72-Bfa1 and Spc72-Bub2 chimeric proteins were functional based on their ability to complement lack of endogenous *BFA1* or *BUB2*, respectively, for what concerns the checkpoint response to microtubule depolymerisation. Indeed, in the presence of nocodazole, *SPC72-BFA1 and SPC72-BUB2* cells arrested in mitosis with 2C DNA contents as well as wild type cells, whereas *bub2Δ* and *bfa1Δ* cells re-replicated their genome in the same conditions (Fig. 2C-D). Thus, the Spc72-Bfa1 and-Bub2 chimera are likely functional in that they retain their inhibitory properties towards Tem1.

Previously characterized Bub2 and Bfa1 chimeric proteins constitutively anchored to SPBs are SPOC-defective [36]. Similarly, our Spc72-Bfa1 and Spc72-Bub2 failed to activate the SPOC upon spindle mispositioning caused by *DYN1* deletion (Fig. 2E). Indeed, *dyn1Δ SPC72-BFA1 bfa1Δ*cells undergoing anaphase in the mother cell, which is symptomatic of spindle mispositioning, exited the cell cycle and re-budded, in contrast to *dyn1Δ* cells that arrested in mitosis as large budded cells (Fig. 2E). The SPOC failure of *SPC72-BFA1 bfa1Δ* cells was not worsened by deletion of *BUB2* or *KIN4* or both, consistent with the notion that Kin4 and Bub2/Bfa1 act in concert to inhibit Tem1. Similar results were obtained with the Spc72-Bub2chimera (S1 Fig.). Thus, constitutive targeting to both SPBs of the GAP Bub2/Bfa1, and of Tem1 as a consequence, leads to unscheduled Tem1 activation, consistent with a previous proposal [22].

We then asked if symmetric localization of Tem1 driven by the Spc72-Bfa1 chimera leads to more efficient recruitment of Cdc15 to SPBs in metaphase. This was indeed the case. Whereas GFP-tagged Cdc15 was present at the SPBs of 55% wild type metaphase cells, 90% of metaphase cells expressing the fusion *SPC72-BFA1* displayed SPB-bound Cdc15 (Fig. 2F). Furthermore, Cdc15 was significantly more symmetric in *SPC72-BFA1* than in wild type cells. Thus, stable tethering of Tem1 to SPBs by fusion to an SPB component [22] or by SPB recruitment via its inhibitory GAP ([36] and our data) leads in both cases to premature Tem1 activation.

We then asked if expression of the Spc72-Bfa1 chimera could have any phenotypic consequence for conditional mutants affecting the MEN. Remarkably, *SPC72-BFA1* as the only source of Bfa1 in the cells suppressed the growth defects of several MEN mutants. In particular, it could partially rescue the temperature sensitivity of *tem1–3* and *mob1–77*(Fig. 2G), as well as the cold-sensitivity of *cdc15–2* and *dbf2–2* mutant cells (S2A Fig.). A slight suppression, if any, was observed for the temperature-sensitivity of *cdc5–2* cells, whereas the temperature-sensitivity of *cdc14–3* cells was not suppressed at all (S2B-C Fig.).

Suppression of *tem1–3* was recessive, as it was not observed when a wild type copy of *BFA1* was present concomitant to *SPC72-BFA1* in the cells (Fig. 2G). Importantly, suppression was not due to reduced GAP activity, as it could not be recapitulated by deletion of *BFA1*(Fig. 2G), or *BUB2* or both (S2D Fig.). Since the mitotic exit defects of *tem1–3* cells at high temperatures correlate with a loose interaction of the mutant Tem1 protein with SPBs [53], we conclude that Spc72-Bfa1 suppresses the temperature-sensitivity of *tem1–3* cells likely by recruiting Tem1 to the SPBs. Consistent with this notion, the Spc72-Bfa1 and Spc72-Bub2 chimera suppressed the lethality caused by overexpression of the SPOC kinase Kin4 (Fig. 2H), which increases the turnover of Bub2/Bfa1, and by consequence of Tem1, at SPBs [36].

Thus, these data, together with those from a previous study [36], indicate that symmetric persistence of Bub2/Bfa1 at SPBs does not interfere with mitotic exit. Rather, in spite of being part of an inhibitory GAP complex, Bfa1 is a receptor of Tem1 at SPBs, where Tem1 promotes MEN signaling. Stable residence of Bub2/Bfa1 at SPBs causes unscheduled mitotic exit by decreasing Tem1 turnover at SPBs, as previously suggested [22].

### Activation of the FEAR pathway in anaphase is required for the unscheduled mitotic exit triggered by Spc72-Bfa1 and-Bub2 chimeric proteins

**Fig 3:**
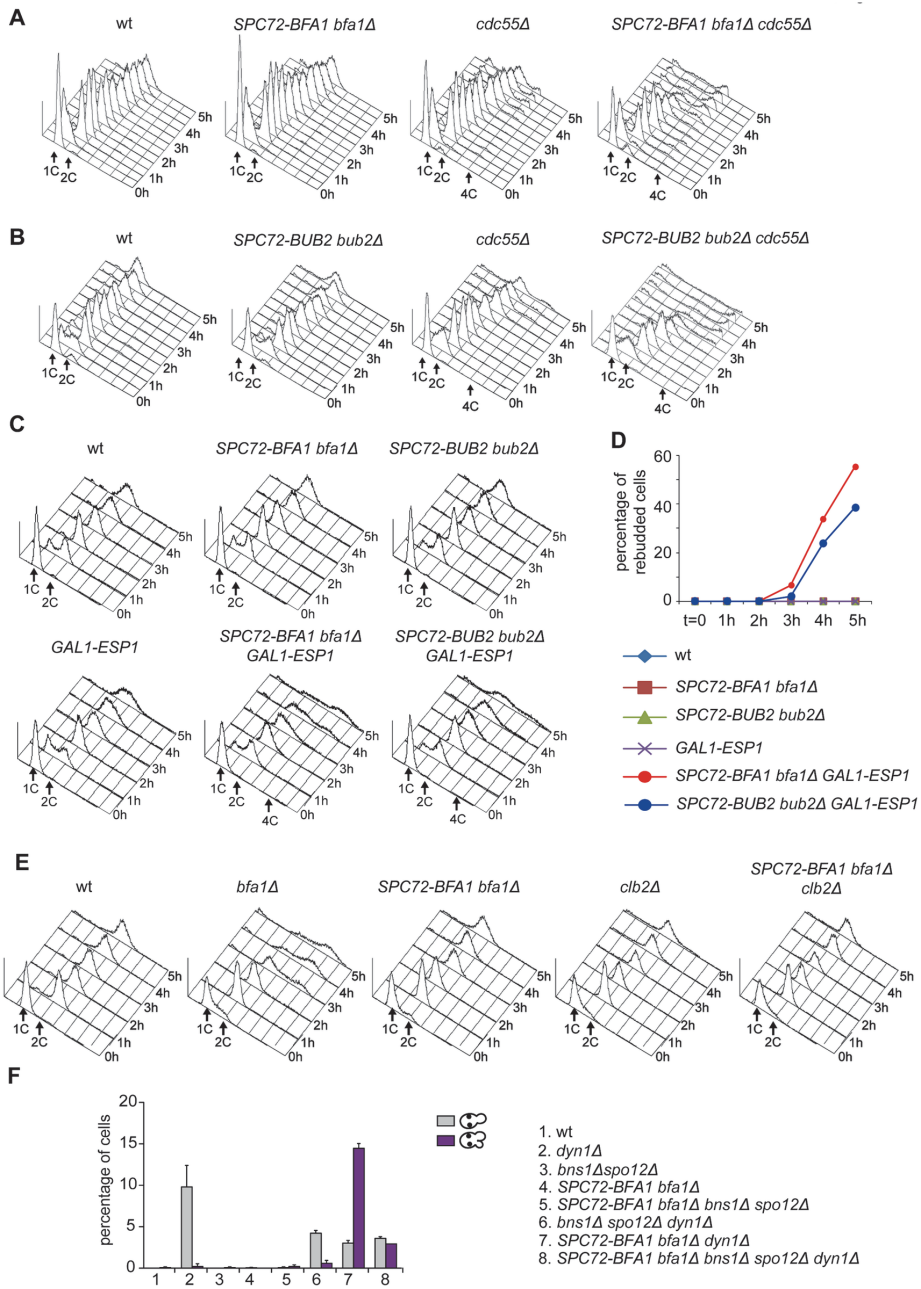
The activation state of the FEAR pathway influences the ability of Spc72-Bfa1 to promote unscheduled mitotic exit. A-E: Logarithmically growing cultures of cells with the indicated genotypes were synchronized in G1 by α-factor and then released into nocodazole-containing medium (t = 0). At the indicated times cell samples were withdrawn for FACS analysis of DNA contents (A-C, E) and to score the percentage of re-budded cells (D). F: The percentage of cells with binucleate cell bodies accompanied or not by a SPOC defect was scored after DAPI staining of cycling cells with the indicated genotypes shifted to14°C for 16h.

The ability of Spc72-Bfa1 and-Bub2 tethers to efficiently prevent mitotic exit upon microtubule depolymerization, but not upon spindle mispositioning, was somewhat puzzling. A major difference between the two conditions lies in the activation of the spindle assembly checkpoint (SAC) after nocodazole treatment. Through inhibition of Cdc20/APC, SAC leads to securin stabilization, in turn preventing activation of separase and the FEAR pathway [15]. If inhibition of the FEAR pathway is the only reason for the failure of Spc72-Bfa1 and-Bub2 chimera to promote mitotic exit, premature FEAR activation should allow mitotic exit in cells expressing Spc72-Bfa1 and-Bub2 treated with nocodazole. Conversely, FEAR inactivation should prevent mitotic exit in the same cells undergoing spindle misalignment. To test this hypothesis, we prematurely activated the FEAR pathway and Cdc14 release from the nucleolus by either inactivation of the PP2A^Cdc55^ phosphatase or *ESP1* overexpression [16]. Remarkably, PP2A^Cdc55^ inactivation through deletion of *CDC55* had a synergistic effect with *SPC72-BFA1* and *SPC72-BUB2* on the kinetics of mitotic exit upon nocodazole treatment, as judged by the ability of cells to re-replicate their DNA (Fig. 3A-B). Similar data were obtained with *ESP1* overexpression from the galactose-inducible *GAL1* promoter (Fig. 3C-D). In contrast, reducing the levels of mitotic CDKs through *CLB2* deletion did not accelerate mitotic exit in *SPC72-BFA1* cells (Fig. 3E). Importantly, FEAR inactivation through deletion of both *SPO12* and *BNS1* reduced the unscheduled mitotic exit caused by *SPC72-BFA1* in *dyn1Δ* cells (Fig. 3F).

Thus, constitutive recruitment of the Bub2/Bfa1 complex to SPBs leads to precocious Tem1 activation. Whether this translates into a premature mitotic exit depends on the activation state of the FEAR pathway.

### A constitutively active GTP-bound Tem1 variant is SPOC-deficient and is synthetically lethal for mutants affecting spindle positioning

**Fig 4:**
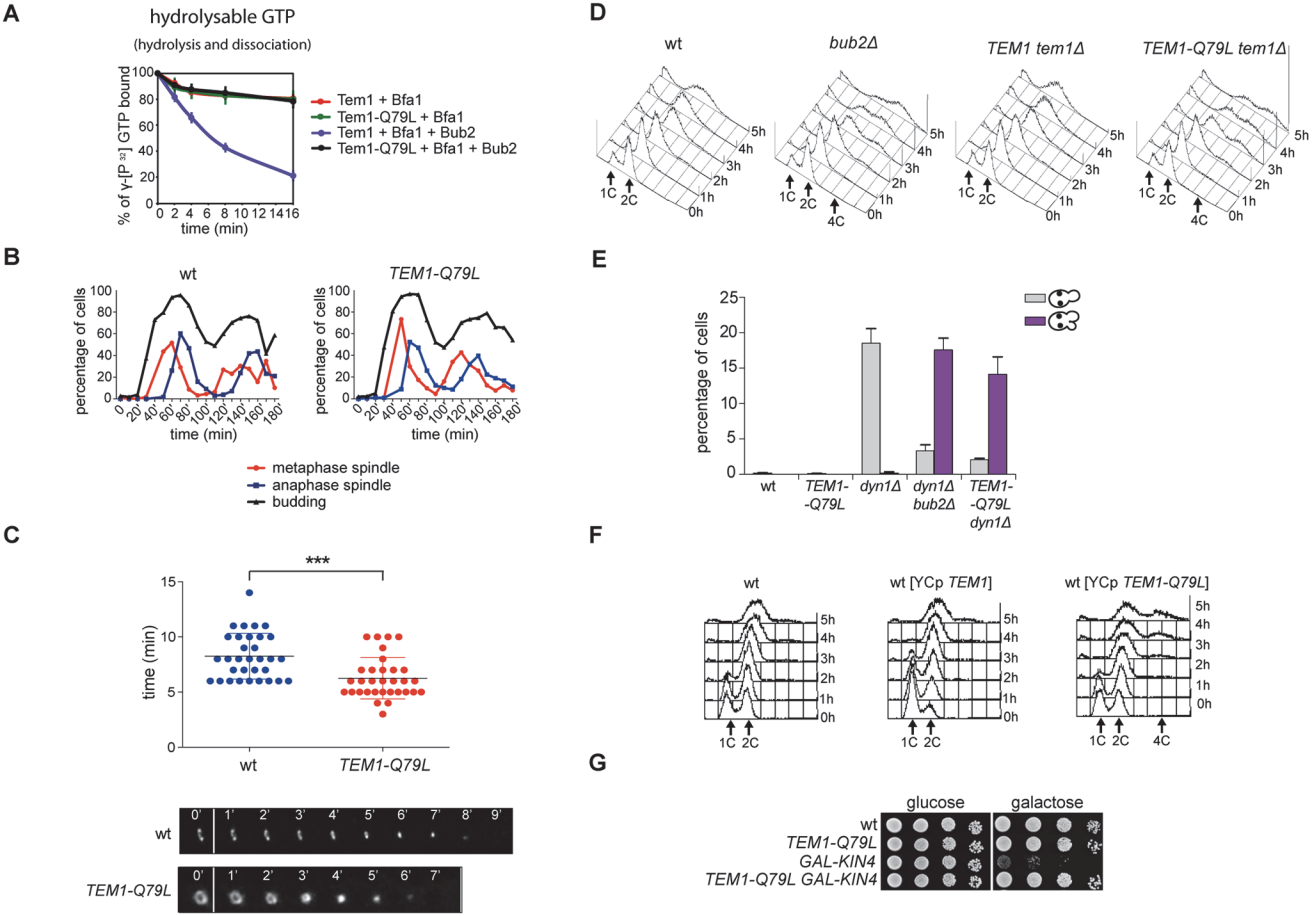
The constitutively active Tem1-Q79L variant is checkpoint-deficient. A: Bacterially purified 6XHis-Tem1 and 6XHis-Tem1-Q79L were loaded with γ[^32^P]GTP either in the absence or in the presence of recombinant MBP-Bfa1 and incubated at 30°C for 10 minutes. The mixture was then added to GST-Bub2 or buffer alone and kinetics of GTP hydrolysis and dissociation was followed by a filter-binding assay (see details in Material and Methods). Graphs show average values and standard deviations from three independent experiments. B: Wild type and *TEM1-Q79L* cells were arrested in G1 by α-factor and then released into fresh medium at 25°C (t = 0). Cell samples were withdrawn every 10’ to measure kinetics of budding and spindle formation/elongation after *in situ* immunostaining of tubulin. C: Actomyosin ring contraction has been visualized by live cell imaging of wild type and *TEM1-Q79L* expressing Myo1-GFP (n = 30). D: Logarithmically growing cultures of cells with the indicated genotypes were shifted into nocodazole containing medium (t = 0). DNA contents were analysed by flow cytometry at the indicated times. E: The percentage of cells with binucleate cell bodies accompanied or not by SPOC defect was scored after DAPI staining of cycling cells of the indicated strains shifted to 14°C for 16h. F: Logarithmically growing cultures of strains with the indicated genotypes were shifted to nocodazole containing medium (t = 0). DNA contents were analysed by flow cytometry at the indicated times. G: Serial dilutions of stationary phase cultures of the indicated strains were spotted on YPD or YP galactose plates and incubated at 30°C for 48h.

To further investigate the links between Tem1 activity and the establishment of SPB asymmetry of the Tem1/Bub2/Bfa1 complex, we generated a *TEM1-Q79L* mutant allele, where the catalytic glutamine in the G domain (Q79, according to sequence comparison with Rab-like GTPases [27]), was replaced by leucine. We first tested the catalytic properties of Tem1-Q79L in *in vitro* GTPase assays in the presence of Bfa1 and Bub2. As shown in Fig. 4A, Tem1-Q79L was completely refractory to stimulation of GTP hydrolysis by the GAP Bub2/Bfa1 *in vitro*, suggesting that *in vivo* it is preferentially in its active GTP-bound form. Thus, Q79 of Tem1 likely participates directly to GTP hydrolysis along with R85 and Q132 of Bub2.

When expressed in yeast cells as the sole source of Tem1, Tem1-Q79L did not cause any detectable growth defect at any temperature. Furthermore, *TEM1-Q79L* mutant cells showed kinetics of cell cycle progression similar to those observed in wild type cells (Fig. 4B). The absence of obvious cell cycle phenotypes in unperturbed conditions was somewhat surprising, as a similar mutation in fission yeast *spg1+*, encoding the SIN counterpart of the Tem1 GTPase, leads to premature cytokinesis and formation of multiple septa [54]. We therefore analysed by time-lapse video microscopy the speed of actomyosin ring contraction, as a marker of cytokinesis [55], in wild type and *TEM1-Q79L* cells expressing GFP-tagged myosin II (Myo1). Strikingly, contraction of the actomyosin ring took place on average 2’ faster in *TEM1-Q79L* relative to wild type cells (i.e. 6’ and 8’, respectively, Fig. 4C), consistently with previous data on *bub2Δ* cells [56]. Thus, the *TEM1-Q79L* allele accelerates at least some aspects of cytokinesis without affecting cell viability.

To gain further insights into the factors allowing *TEM1-Q79L* cells to grow at normal rates, we carried out a synthetic genetic arrays (SGA) screen to find deletions of non-essential genes that become synthetically lethal/sick with *TEM1-Q79L*(Table 1). This screen uncovered several genes encoding proteins involved in spindle positioning and nuclear migration, such as Kar9, the dynein light chain (*DYN2*) and components of the dynactin complex that cooperates with dynein for spindle positioning [57]. In addition, this screen uncovered several genes implicated in microtubule biogenesis, which might indirectly influence spindle positioning. Thus, *TEM1-Q79L* aggravates the sickness of cells undergoing spindle mispositioning. Interestingly, the same deletion mutants were also identified in other SGA screens as synthetically sick or lethal with *BUB2* or *BFA1* deletion [58–60]. A number of additional non-essential genes whose deletion displayed synthetic interactions with *TEM1-Q79L* was also uncovered with this screen and will be described elsewhere, since the significance of these genetic interactions has not been further explored in this context.

**Table 1:** List of non-essential genes implicated in microtubules dynamics or spindle positioning identified in the SGA screen with *TEM1-Q79L*.

**GENE**	**DESCRIPTION**	**FUNCTION**
***BIK1***	Microtubule-associated protein	Spindle positioning and nuclear migration
***NUM1***	Nuclear migration	Spindle positioning and nuclear migration
***BIM1***	Microtubules plus end-tracking protein	Spindle positioning and nuclear migration
***DYN2***	Dynein light chain	Spindle positioning and nuclear migration
***ARP1***	Component of the dynactin complex	Spindle positioning and nuclear migration
***LDB18***	Component of the dynactin complex	Spindle positioning and nuclear migration
***NIP100***	Component of the dynactin complex	Spindle positioning and nuclear migration
***JNM1***	Component of the dynactin complex	Spindle positioning and nuclear migration
***CIK1***	Kinesin-associated protein	Spindle positioning and nuclear migration
***KAR9***	Microtubule-associated protein	Spindle positioning and nuclear migration
***PAC10***	Co-chaperone GimC/prefoldin complex	Microtubules biogenesis
***GIM3***	Co-chaperone GimC/prefoldin complex	Microtubules biogenesis
***YKE2***	Co-chaperone GimC/prefoldin complex	Microtubules biogenesis

We then tested the ability of *TEM1-Q79L* mutant cells to respond to microtubule depolymerization and spindle mispositioning. In the presence of nocodazole, whereas wild type cells arrested in mitosis with 2C DNA contents, *TEM1-Q79L* cells re-replicated their genome similar to cells lacking Bub2 (Fig. 4D). In addition, *TEM1-Q79L dyn1Δ* cells exited mitosis and re-budded in face of spindle position defects (Fig. 4E). Thus, the *TEM1-Q79L* mutant allele affects SPOC response. As expected, the checkpoint defect was dominant, as the *TEM1-Q79L* allowed mitotic exit and re-replication in the presence of nocodazole even when expressed from an episomal plasmid in cells carrying also the endogenous *TEM1* gene (Fig. 4F). Consistent with its constitutive activation, the *TEM1-Q79L* allele was also able to suppress the lethality associated with overexpression of *KIN4* from the galactose-inducible *GAL1* promoter (Fig. 4G), which delays mitotic exit by keeping the Bub2/Bfa1 GAP active [34].

### The constitutively active Tem1-Q79L variant shows reduced asymmetry at spindle poles and impairs Bfa1 asymmetry

**Fig 5:**
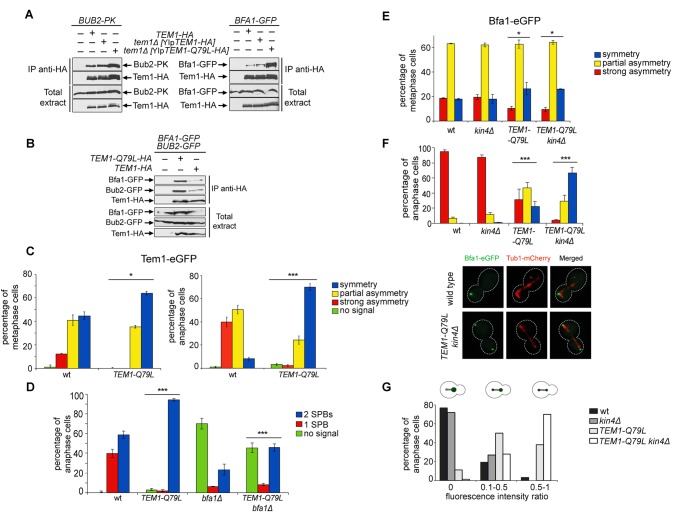
Constitutively active Tem1-Q79L binds more avidly Bub2/Bfa1 and shows reduced asymmetry at anaphase spindle poles. A-B: Protein extracts from cells expressing the indicated tagged proteins were used for immunoprecipitation with an anti-HA affinity resin. Western blot analysis was then performed with anti-PK, anti-GFP and anti-HA antibodies. The input represents 1/25^th^ of the total extract used for each IP. C-F: Localization of eGFP- tagged Tem1 and Tem1-Q79L (C-D) or Bfa1-eGFP (E-F) was analysed in the indicated strains by fluorescence microscopy after formaldehyde fixation. Metaphase and anaphase cells were identified by means of the Tub1-mCherry co-expressed marker. Micrographs show representative cells of each strain in anaphase. G: Fluorescence intensity ratios were calculated between the two SPBs in anaphase cells of the indicated strains (see details in Materials and Methods).

The “dual finger” model predicts that GTP hydrolysis is catalysed by the GAP and the so-called catalytic glutamine of the GTPase could stabilize the interaction between the GAP and the GTPase without directly contributing to the catalytic reaction [48]. We formally tested this idea by analysing the interaction between Tem1 or Tem1-Q79L with Bub2 and Bfa1 in co-immunoprecipitation experiments. Remarkably, HA-tagged Tem1-Q79L pulled down a higher, rather than a lower, amount of Bub2 and Bfa1 (tagged with 3PK epitopes and GFP, respectively) (Fig. 5A). Thus, constitutive binding to GTP seems to increase the affinity of Tem1 for its GAP. To further corroborate this conclusion we co-expressed Bfa1-GFP and Bub2-GFP with HA-tagged Tem1 or Tem1-Q79L. Immunoprecipitation of Tem1-Q79L-HA3 pulled down higher amounts of both Bfa1-GFP and Bub2-GFP than Tem1-HA3 (Fig. 5B), without affecting the relative proportion of Bfa1-GFP and Bub2-GFP in the immunoprecipitates. We therefore conclude that locking Tem1 in the GTP-bound form enhances its affinity for Bub2/Bfa1 without affecting the stoichiometry of the GAP complex.

Since our data suggest that proper regulation of Tem1 GTP hydrolysis is required for asymmetry of the Tem1/Bub2/Bfa1 complex at SPBs, we analysed the subcellular distribution of Tem1-Q79L tagged with eGFP. As expected, Tem1-eGFP was already asymmetric in 53% of metaphase cells, whereas Tem1-Q79L-eGFP was asymmetric in a lower fraction of cells (35%). In anaphase, whereas Tem1-eGFP localized asymmetrically to the bud-directed SPB in 90% of cells, Tem1-Q79L-eGFP was present symmetrically at SPBs in 70% of the cells (Fig. 5C). Thus, abolishing the GAP-stimulated GTPase activity of Tem1 through different kinds of mutations invariably leads to Tem1 symmetric localization at SPBs.

Interestingly, whereas *BFA1* deletion markedly affected Tem1-eGFP recruitment to SPBs as previously reported [22, 40, 61], it had a less pronounced effect on the SPB localization of Tem1-Q79L-eGFP (Fig. 5D), suggesting that loading to SPBs of constitutively active Tem1 is partially GAP-independent.

Previous data suggested that loading of Bfa1 and Bub2 on SPBs and their asymmetry in anaphase still occur in cells lacking Tem1 [30, 40]. On the other hand, experimental evidence indicates that an increased residence time of Tem1 at the SPBs or its decreased GTPase activity can influence Bub2/Bfa1 localization [22, 25, 62]. Because our results indicate that the Q79L substitution affects Tem1 activity as well as its localization in anaphase, we asked if Tem1-Q79L had any impact on localization of Bfa1. Both wild type and *TEM1-Q79L* mutant cells showed a similar partially asymmetrical SPB localization of Bfa1-eGFP in metaphase (Fig. 5E). In contrast, at the onset of anaphase, while Bfa1 drastically dropped to hardly detectable levels on the mother-bound SPB in 95% of wild type cells, it remained completely symmetrical on SPBs in 22% of *TEM1-Q79L* cells and persisted to low but clearly detectable levels on the mother-bound SPB in 46.6% of the cells (Fig. 5F). The symmetric localization of Bfa1 in *TEM1-Q79L* cells did not depend on premature activation of downstream MEN kinases, as it was not affected by Cdc15 inactivation through the *cdc15–2* temperature-sensitive allele (S3 Fig.).

Since Kin4 promotes Bfa1 turnover at SPBs upon SPOC activation [36], we analysed Bfa1 localization in wild type and *TEM1-Q79L* cells lacking *KIN4*(Fig. 5E-F). In agreement with previous data [36], deletion of *KIN4* alone did not affect Bfa1 distribution on SPBs of wild type cells in unperturbed conditions. In stark contrast, it had a synergistic impact with the *TEM1-Q79L* mutant allele on Bfa1 localization at SPBs specifically in anaphase, making it completely symmetrical in 65% of the cells and partially asymmetric in 30% of the cells (Fig. 5F). Consistently, the ratio in Bfa1-GFP fluorescence intensity at the mother- versus the bud-directed SPB was close to 0 for wild type and *kin4Δ* cells, while it significantly increased in *TEM1-Q79L* and *TEM1-Q79L kin4Δ* cells (Fig. 5G). Thus, these data reveal an unanticipated role of Kin4 in actively dislodging Bfa1 from the mother-bound SPB during anaphase of the unperturbed cell cycle. Furthermore, they indicate that Tem1 GTP hydrolysis is a primary determinant of Bfa1 asymmetry in anaphase.

### The *TEM1-Q79L* allele enhances Cdc15 loading on SPBs, without affecting the timing of mitotic exit

**Fig 6:**
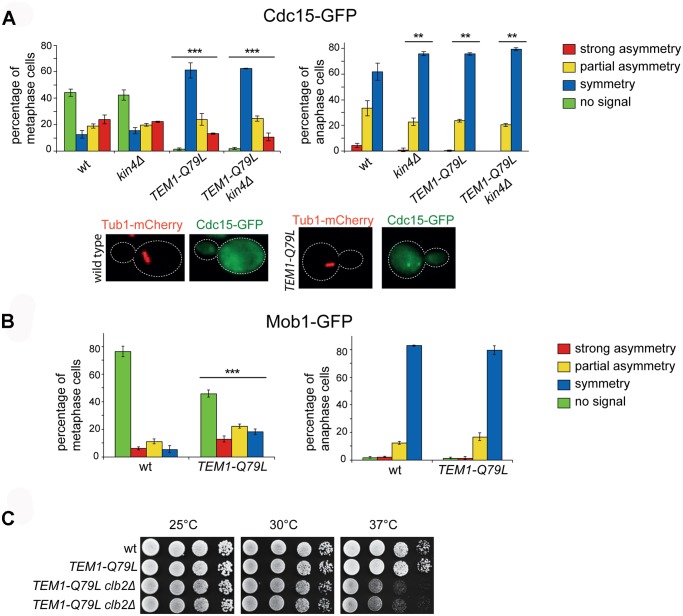
The *TEM1-Q79L* allele leads to premature Cdc15, but not Mob1, loading on SPBs. A-B: Distribution of Cdc15-GFP (A) or Mob1-GFP (B) was analysed in the indicated strains by fluorescence microscopy after formaldehyde fixation. Metaphase and anaphase cells were identified by means of the Tub1-mCherry co-expressed marker. Micrographs show representative wild type and *TEM1-Q79L* cells expressing Cdc15-GFP in metaphase. C: Serial dilutions of stationary phase cells with the indicated genotypes were spotted on YPD and incubated at the indicated temperatures for 48h.

To investigate further if the *TEM1-Q79L* allele leads to premature MEN activation, we analysed the subcellular localization of downstream MEN components, such as Cdc15 and Mob1. We observed that 99% of *TEM1-Q79L* mutant cells recruited Cdc15 to SPBs in metaphase, as opposed to 55% in wild type cells (Fig. 6A). Furthermore, Cdc15 was significantly more symmetric in *TEM1-Q79L* than in wild type cells. Deletion of *KIN4*, either alone or in combination with the *TEM1-Q79L* allele did not have any impact on Cdc15 distribution (Fig. 6A). In spite of Cdc15 enhanced loading on SPBs, recruitment of Mob1 to SPBs in metaphase was only slightly increased in *TEM1-Q79L* relative to wild type cells (Fig. 6B), consistent with the notion that mechanisms other than the SPOC restrain MEN activity downstream of Tem1 until late anaphase.

Phosphorylation of Cdc15 and Mob1 by cyclin B/CDKs together with Bub2/Bfa1 GAP activity provides a dual inhibition of the MEN [63]. Consistently, we found that combining the *TEM1-Q79L* allele with deletion of *CLB2*, which encodes the main mitotic cyclin B, caused synthetic growth defects at 37°C (Fig. 6C). However, no synthetic lethality or detrimental synthetic defects were induced by the additional deletion of *KIN4* or *BFA1*, suggesting that the components of the MEN downstream of Cdc15 are likely targets of negative regulators additional to Bub2/Bfa1 and Clb2-associated CDKs.

### Bub2/Bfa1 and Tem1 asymmetry is important for proper Kar9 distribution and spindle positioning

**Fig 7:**
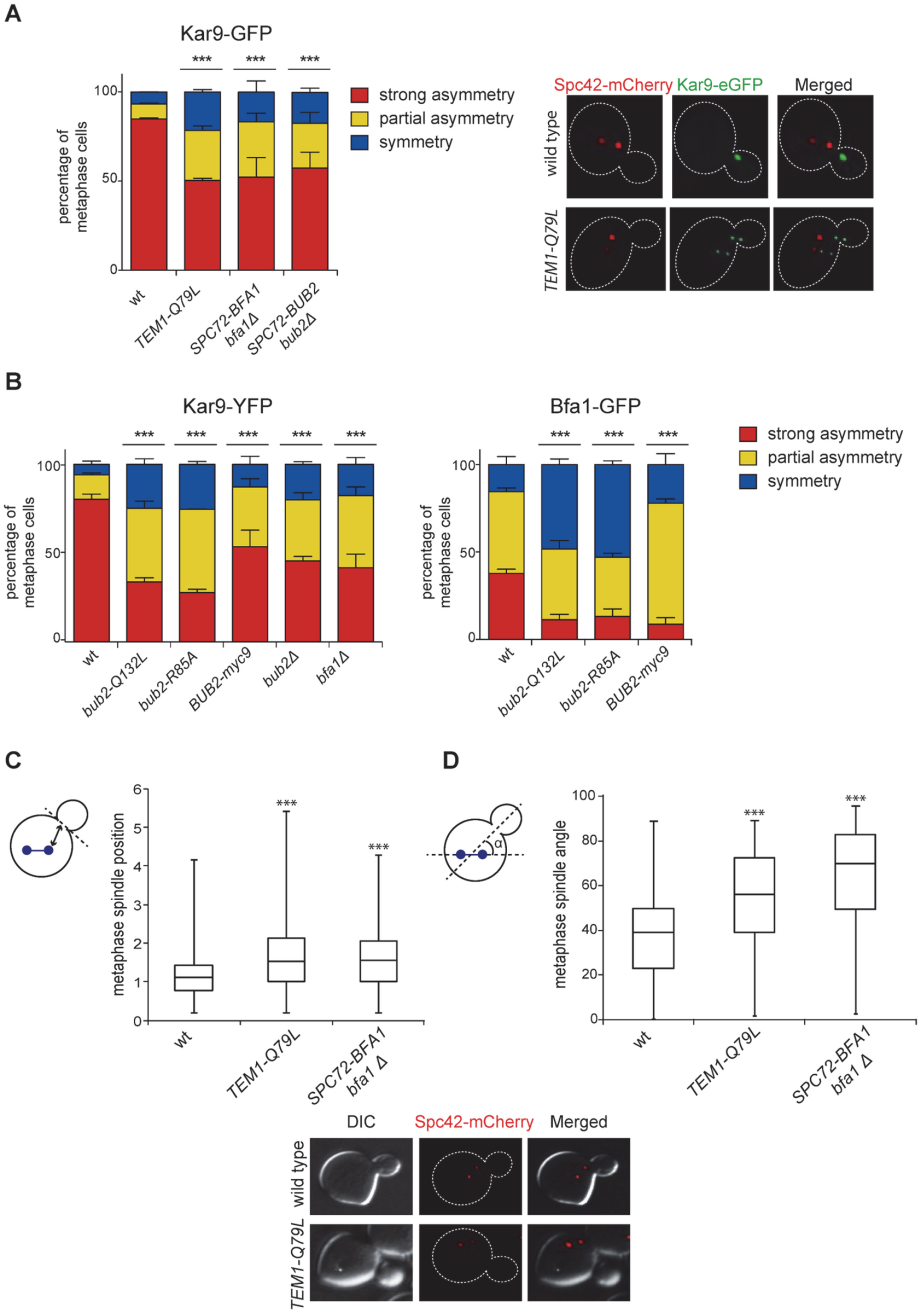
Bub2/Bfa1 and Tem1 asymmetry is important for proper Kar9 distribution and spindle positioning. A-B: Percentage of metaphase cells carrying Kar9-eGFP (A), Kar9-YFP or Bfa1-eGFP (B) on one SPB (strong asymmetry), both SPBs unequally (partial asymmetry) or both SPBs equally (symmetry) for the indicated strains after formaldehyde fixation. Metaphase and anaphase cells were identified by means of the co-expressed SPB marker Spc42-mCherry. C-D: Spindle position (distance between the nearest SPB and the bud neck) and orientation (angle that the spindle forms with respect to the cell polarity axis) were measured in metaphase spindles of cells with the indicated genotypes after formaldehyde fixation (n≥100).

Partial asymmetry of Bub2/Bfa1 at SPBs begins already in metaphase [39]. Since during metaphase MEN induces asymmetric localization of Kar9 at spindle poles, which is in turn required for correct spindle positioning [18], we checked if symmetrical Bub2/Bfa1 and Tem1 might affect Kar9 localization and spindle positioning. To this end, we analysed the distribution of Kar9 tagged with eGFP on the metaphase spindles of wild type, *SPC72-BFA1 bfa1Δ, SPC72-BUB2 bub2Δ*, and *TEM1-Q79L* cells. Whereas 84.4% of wild type cells showed strongly asymmetric Kar9, this value dropped to 43.5%, 50.5% and 47.6% in *SPC72-BFA1 bfa1Δ, SPC72-BUB2 bub2Δ* and *TEM1-Q79L* cells, respectively, while the remaining fraction of cells displayed partial or complete symmetry (Fig. 7A).

**Fig 8:**
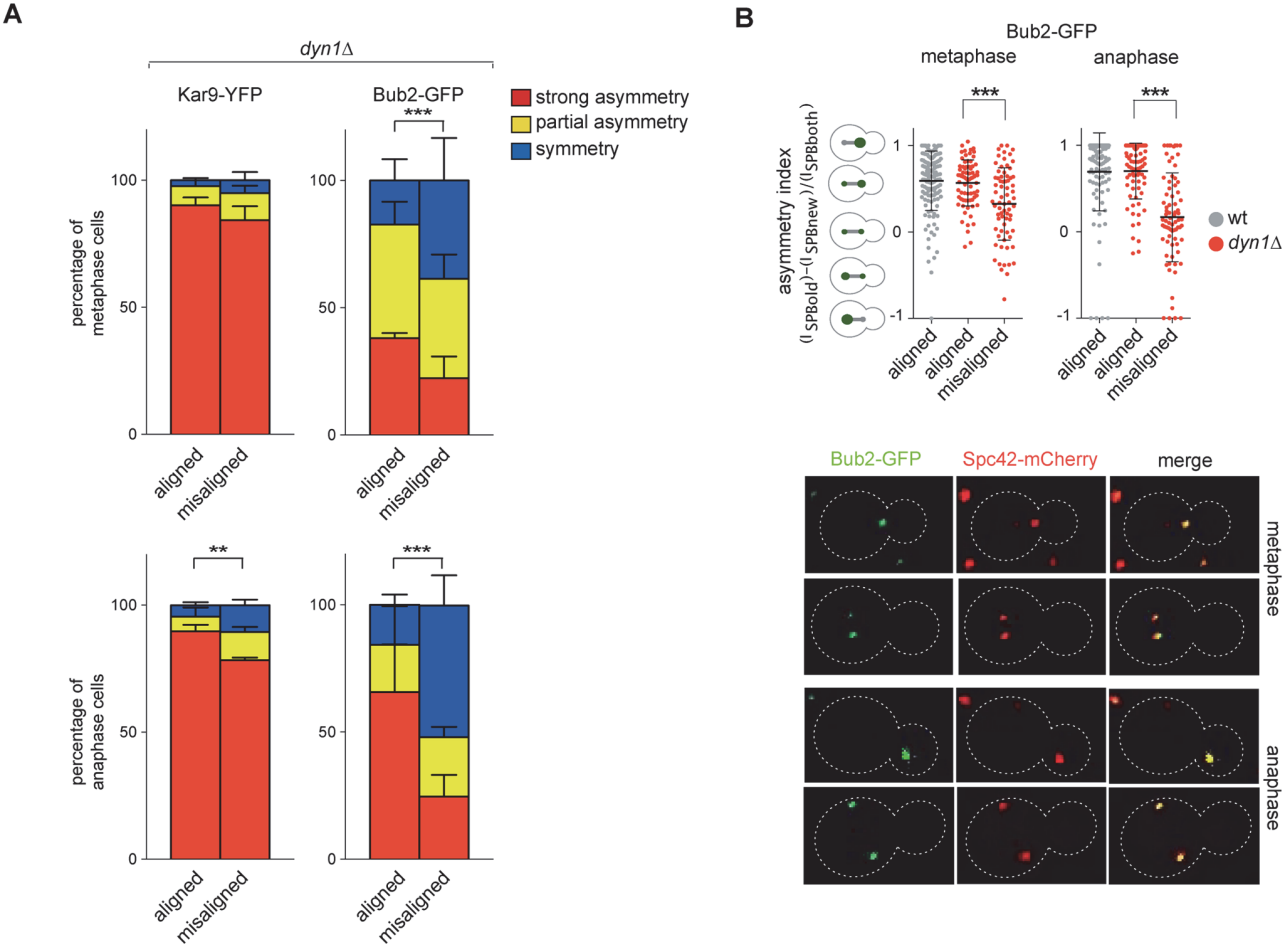
Bub2, but not Kar9, become symmetric on misaligned spindles. **A**: Distribution of Kar9-YFP and Bub2-GFP in metaphase and anaphase on aligned and misaligned (*dyn1Δ*) spindles. Metaphase and anaphase cells were identified by means of the co-expressed SPB marker Spc42-mCherry. Misaligned spindles in metaphase cells were identified by measuring an angle of 60°-120° between the spindle axis and a line connecting the old SPB (defined as the SPB showing brighter Spc42-mCherry fluorescence) to the bud neck. Scoring was done on 3 independent clones for each genotype with a total N ≥ 75; mean ± SD. **B**: Asymmetry indices were calculated for wild type and *dyn1Δ* cells expressing Bub2-GFP and Spc42-mCherry in (A) by dividing the difference between Bub2 fluorescence associated with the old SPB (I_SPBold_) and new SPB (I_SPBnew_) by the total Bub2 fluorescence (I_SPBboth_). Age of SPBs was determined by the fluorescence intensity of Spc42-mCherry (bright fluorescence: old SPB, dim fluorescence: new SPB). Micrographs show representative Bub2-GFP distribution on aligned and misaligned spindles, in either metaphase or anaphase.

Since our results indicate that the GAP activity of Bub2 and Bfa1 influences the localization of the GAP complex in the cell, we characterized Kar9 distribution in the GAP-dead Bub2 variants, *bub2-Q132L* and *bub2-R85A* cells, in cells expressing the symmetric GAP-inactive *BUB2-myc9* construct [25]. All strains showed more symmetric localization of Kar9, with 33.9% of complete asymmetry for *bub2-Q132L*, 27.8% for *bub2-R85A*, 53.6% for *BUB2-myc9*, as opposed to 80.3% for wild type cells (Fig. 7B). Surprisingly, we found increased Kar9 symmetry also in *bub2Δ* and *bfa1Δ* mutant cells (45.7% and 41.9% of complete asymmetry, respectively), where Tem1 is present at SPBs at low levels [22], but symmetrically (Fig. 5C). Increased symmetry of Kar9 in the mutants (with the exception of *bub2Δ* and *bfa1Δ* that were not analysed) was accompanied by increased Bfa1 symmetry (Fig. 7B). Therefore, establishment of Bub2/Bfa1 and Tem1 asymmetry impacts on Kar9 asymmetry. Nonetheless, Bub2/Bfa1 symmetric distribution at SPBs is not sufficient to drive Kar9 symmetry. Indeed, when spindles were misaligned in *dyn1Δ* mutant cells Bub2 became increasingly more symmetric from metaphase to anaphase, whereas Kar9 symmetry increased only slightly (Fig. 8A-B), in agreement with recently published data [64, 65]. Thus, once Kar9 asymmetry has been established, it cannot be reversed by spindle misalignment, in contrast to that of Bub2/Bfa1.

We then asked if Kar9 mislocalization in our mutants affects spindle positioning. *SPC72-BFA1* and *TEM1-Q79L* cells expressing Spc42-mCherry were synchronized to collect cells in metaphase and imaged. Measurements of spindle distances from the bud neck (Fig. 7C) and spindle angles relative to the mother-bud polarity axis (Fig. 7D) indicated that both *TEM1-Q79L* and *SPC72-BFA1* cells significantly affected the position and the orientation of metaphase spindles. Thus, hyperactive Tem1 impaired Kar9-dependent spindle positioning.

In conclusion, although constitutive symmetric localization of Tem1 and its GAP Bub2/Bfa1 does not appear to affect mitotic exit, it does compromise asymmetry of Kar9 at spindle poles, thereby causing spindle positioning and orientation defects.

## Discussion

### Tem1 GTP hydrolysis involves a catalytic glutamine in the switch II and the GAP Bub2/Bfa1

The mechanistic details of how GTPases switch between a GTP- and a GDP-bound state build on initial structural studies on Ras. In Ras a conserved glutamine in the switch II domain of the GTPase and a conserved arginine of the GAP both contribute to GTP hydrolysis [66, 67] and, consistently, mutations of either residue abolish GTP hydrolysis. Crystal structure of some Rab GTPases in complex with their TBC (Tre-2, Bub2 and Cdc16) GAP revealed that the conserved switch II glutamine (Q79 of Tem1) does not directly participate in GTP hydrolysis. Rather, catalysis is entirely brought about by a conserved arginine and a conserved glutamine of the TBC GAP through a mechanism referred to as “dual-finger” [48, 68]. Hence, the switch II glutamine of the GTPase was proposed to stabilize its interaction with the GAP [48]. Recently, however, Rab GTPases have been shown to be more plastic than originally anticipated in their activation/hydrolysis mechanisms. In particular, the contribution of the switch II glutamine in GTP hydrolysis is variable and for some GTPases it contributes, together with a conserved lysine in the P-loop, to activation of the GTPase by stabilizing its GEF-bound nucleotide-free form [69]. Therefore, the outcome of mutations of conserved catalytic residues varies depending on the GTPase, GEF and GAP, and is altogether unpredictable.

Here we have addressed the importance of Q79 in the switch II and the dual-finger mechanism in Tem1 GTP hydrolysis. First, we have established that the intrinsic rate of Tem1 GTP hydrolysis is negligible, and loss of GTP is mostly accounted for by nucleotide dissociation. In agreement with previous data [25, 26, 30], Bfa1 prevents nucleotide dissociation and therefore acts as guanine-nucleotide dissociation inhibitor (GDI). Second, we show for the first time that Tem1 Q79 is directly involved in the GAP-induced GTP hydrolysis without impairing its interaction with Bub2 and Bfa1. Mutation of Q79 into leucine generates a hyperactive, dominant Tem1 that is refractory to its GAP, recruits more efficiently Cdc15 to SPBs and leads to unscheduled mitotic exit in the presence of spindle positioning defects.

Finally, we show that the dual-finger mechanism applies also to GTP hydrolysis of the Tem1-Bub2-Bfa1 complex. Indeed, glutamine 132 of Bub2 is involved in GTP hydrolysis, in addition to arginine 85 that we previously showed [25]. Consistent with an important role of Q132 in Tem1 inhibition, *bub2-Q132L* mutant cells are SPOC-defective and undergo mitotic exit upon microtubule depolymerization.

Thus, we have defined Q79 of Tem1 together with R85 and Q132 of Bub2 as a catalytic triad for GAP-induced GTP hydrolysis.

### Asymmetry of Bub2/Bfa1 and MEN activation

The Bub2/Bfa1 complex is required for efficient Tem1 binding to SPBs throughout most of the cell cycle, except in late mitosis [22, 40, 61]. In contrast, SPB recruitment of Bub2/Bfa1 does not require Tem1 [30, 40]. The amount of Tem1 at SPBs depends on the turnover of Bub2/Bfa1 at SPBs, which in turn is accelerated by spindle mispositioning through Kin4-dependent phosphorylation of Bfa1 [36, 39]. Thus, as recently proposed [61], the GAP Bub2/Bfa1 is a major Tem1 receptor at SPBs and its regulation is instrumental for establishing Tem1 asymmetry. Critical regulators of Bub2/Bfa1 asymmetry are the polo kinase Cdc5 and the phosphatase PP2A^Cdc55^. Cdc5 phosphorylates and inactivates the Bub2/Bfa1 complex leading to Tem1 activation [51, 70], whereas PP2A^Cdc55^ dephosphorylates Bfa1 [71]. Phosphomimetic mutations in some Cdc5-dependent Bfa1 phosphorylation sites, as well as loss of PP2A^Cdc55^, are sufficient to induce premature asymmetry of Bub2/Bfa1 at SPBs, whereas Cdc5 inactivation or phospho-ablating mutations in Bfa1 lead to its persistent symmetry [62, 71]. Similar mechanisms might be operational in fission yeast to establish SIN asymmetry. Indeed, polo kinase has been proposed to phosphorylate the Bfa1 homolog Byr4 and promote its dissociation from SPBs [72], thereby influencing the distribution of GTP-bound Spg1 and its effector kinase Cdc7. Furthermore, PP2A regulates Byr4 asymmetry through dephosphorylation of the SIN anchor at SPBs Cdc11 [73, 74]. Indeed, Byr4 binds more efficiently to dephosphorylated Cdc11 [73], whereas the Cdc15-like kinase Cdc7 binds preferentially phosphorylated Cdc11 [75]. Thus, *S. cerevisiae* and *S. pombe* might adopt common regulatory strategies to establish MEN and SIN asymmetry.

We previously proposed that Tem1 GTP hydrolysis promotes asymmetry of both Tem1 and its GAP Bub2/Bfa1 at SPBs in anaphase [25] and results from other studies [22, 62] support this conclusion. Consistent with this idea, we now show that mutating the second catalytic finger of Bub2 (Q132) or mutating the catalytic Q79 of Tem1 leads in both cases to increased symmetry of Bub2/Bfa1 and Tem1 at SPBs. At a first glance these results appear at odds with the finding that in the complete absence of Tem1 Bub2/Bfa1 is not only recruited to SPBs with normal kinetics, but becomes asymmetric in anaphase exactly like in wild type cells [30]. However, we now show that when GTP hydrolysis is abolished Bub2 and Bfa1 bind more avidly to Tem1. Thus, the increased symmetry of Bub2/Bfa1 at SPBs in these conditions likely reflects its stronger affinity for GTP-bound Tem1. The different affinity of Bub2/Bfa1 for GTP- versus GDP-bound Tem1 has important implications for the SPOC, where active Tem1 needs to be quickly inactivated in the presence of a mispositioned spindle.

Based on our previous results using a version of Bub2 tagged with nine myc epitopes at the C-terminus (Bub2-myc9), we proposed that removal of Bub2/Bfa1 from the mother SPB is important for timely mitotic exit [25]. Indeed, Bub2-myc9 is more symmetrically localized at SPBs than wild type Bub2 and is lethal for *cdc5–2* and *tem1–3* mutants because it prevents mitotic exit in these sensitized backgrounds. Now we further tested this idea by expressing chimeric proteins that constitutively recruit the GAP and Tem1 to both SPBs. Contrary to our predictions, these chimeric proteins partially rescued, instead of aggravating, the temperature-sensitive growth phenotype of several MEN mutants. In particular, Spc72-Bfa1 rescued the temperature-sensitivity of *tem1–3* cells likely by suppressing the SPB-binding defects of the mutant Tem1–3 protein at high temperature [53]. Importantly, suppression of MEN mutants by our chimeric proteins is not accounted for by their possible impaired GAP activity, because it could not be recapitulated by *BFA1* and/or *BUB2* deletion.

The chimeric proteins Spc72-Bfa1 and-Bub2 caused also unscheduled mitotic exit in the presence of mispositioned spindles, similar to Bub2- and Bfa1-Cnm67 fusion proteins previously characterized [36]. Therefore, despite different parts of Bub2 and Bfa1 are fused to the SPB anchor (the N-terminus in our Spc72- fusions and the C-terminus in the-Cnm67 chimera), constitutive binding of the Bub2/Bfa1 complex to SPBs invariably leads to SPOC defects. Our data are totally consistent with the notion that Tem1 recruitment to SPBs is necessary for its MEN function [22]. In this scenario, SPB-locked Bub2/Bfa1 activates Tem1 by increasing its symmetry and residence time at SPBs, thereby causing unscheduled mitotic exit in the presence of mispositioned spindles. It is worth noting that symmetry of SIN components, such as the Cdc7 kinase, at SPBs also causes unscheduled cytokinesis and repeated rounds of septation [46, 47], suggesting that asymmetry is a common strategy in *S. cerevisiae* and *S. pombe* to restrain MEN/SIN signalling.

The molecular basis for SPB-driven Tem1 activation is not known. It is possible that the GAP Bub2/Bfa1 at SPBs is constitutively kept inactive by Cdc5-mediated phosphorylation, thereby making Tem1 at SPBs refractory to GAP-mediated inhibition. Upon spindle misalignment, Kin4-mediated dislodgement of Bub2/Bfa1 from SPBs becomes essential for Tem1 inhibition in the cytoplasm and SPOC response [36, 61]. An interesting non-mutually exclusive hypothesis is that a putative GEF for Tem1 localizes at SPBs [76]. However, as mentioned above the identity of Tem1 GEF(s) remains elusive.

The finding that the chimeric proteins Spc72-Bfa1 and-Bub2, similar to Bub2- and Bfa1-Cnm67 [36] and Cnm67-Tem1 [22], support a mitotic arrest after microtubule depolymerization, but not after spindle mispositioning, was somehow puzzling. One major difference between the SAC-mediated metaphase arrest and the SPOC-mediated anaphase arrest is that in the latter, but not in the former, the PP2A^Cdc55^ phosphatase is inhibited by the FEAR pathway [15, 16]. We find that, indeed, *CDC55* deletion or *ESP1* overexpression in cells expressing Spc72-Bub2 or-Bfa1 drives unscheduled mitotic exit in the presence of nocodazole. In contrast, FEAR inhibition by deletion of *SPO12* and *BNS1* [15] prevents mitotic exit in cells expressing the same chimeric proteins and experiencing spindle position defects. Thus, whether the FEAR is activated or inhibited influences the impact of the chimeric proteins on mitotic exit. PP2A^Cdc55^ has been recently shown to antagonize the Cdc5-dependent phosphorylation of Bfa1 [71], thereby providing a mechanistic explanation to our data. The FEAR-mediated partial release of Cdc14 from the nucleolus might also contribute to MEN activation by counteracting the CDK-dependent inhibitory phosphorylation of MEN components [63].

In conclusion, persistent symmetric localization of the GAP Bub2/Bfa1 does not interfere with mitotic exit. Most likely, the Bub2-myc9 protein that we described previously prevents timely MEN activation by a different mechanism. Consistently, tagging of Spc72-Bub2 at the C-terminus with nine myc epitopes causes synthetic sickness in combination with the *cdc5–2* mutant allele affecting the polo kinase (S1 Table).

### An unanticipated role of Bub2/Bfa1 and Tem1 asymmetry on Kar9 distribution and spindle positioning

We show that symmetric localization of the Bub2/Bfa1/Tem1 complex, independently of whether it is driven by chimeric proteins or loss of GTPase activity, interferes with Kar9 asymmetry at spindle poles in metaphase, as well as with spindle positioning and orientation relative to the cell division axis. Although Tem1 inactivation causes similar phenotypes for what concerns Kar9 localization and spindle orientation, it does not affect spindle positioning at the bud neck [18], indicating that Tem1 hyperactivation and inactivation are not equivalent in this respect. The molecular bases of this difference remain to be established. Similarly, whether Tem1 hyperactivation primarily affects Kar9 localization and, as a consequence, spindle positioning or vice-versa remains to be investigated. Although the phenotypic analyses of our mutants did not reveal any apparent alterations of astral microtubules, at the moment we cannot exclude that subtle defects in microtubule dynamics could account for spindle mispositioning and, in turn, increased Kar9 symmetry.

Previous [64] and our data indicate that not all conditions leading to symmetric distribution of the Bub2/Bfa1 complex and Tem1 cause symmetric localization of Kar9 at spindle poles. Indeed, whereas Bub2, Bfa1, Tem1 and Kar9 are all asymmetric, to different extents, on metaphase spindles, Bub2, Bfa1 and Tem1 become increasingly more symmetric upon spindle misalignment, while Kar9 remains strongly asymmetric. These data suggest that establishment of Bub2/Bfa1/Tem1 symmetry on misaligned spindles is an active process and Kar9 asymmetry is so robust that once established it cannot be reversed by bringing the Bub2/Bfa1/Tem1 complex to both spindle poles. In contrast, in our mutants Bub2, Bfa1 and Tem1 are more symmetric already in metaphase and might therefore interfere with the establishment of Kar9 asymmetry. Furthermore, we speculate that in these mutants the residence of Tem1 and the GAP Bub2/Bfa1 at SPBs is relatively stable, while these proteins turn over very fast at the SPBs of misaligned spindles [36]. An alternative explanation to our data is that Tem1 hyperactivation, rather than symmetry, is responsible for Kar9 mislocalization and spindle misalignment. Indeed, while in our mutants Tem1 is likely in the active GTP-bound form, it is inactivated and GDP-bound when the SPOC is turned on.

Factors involved in cell polarity were implicated in the asymmetry of Bub2-Bfa1 at spindle poles [25, 39]. Thus, it is tempting to speculate that in budding yeast the role of cell polarity in spindle positioning might be partly exerted through asymmetric localization of the Bub2-Bfa1-Tem1 trimeric complex at spindle poles, which in turn influences Kar9 asymmetry. Remarkably, other eukaryotic cells (i.e. nematodes, flies and mammals) employ heterotrimeric G proteins for spindle positioning during both symmetric and asymmetric cell division (reviewed in [77]. A striking parallel can be drawn between the asymmetric enrichment of their GDIs GPR-1/2, which is controlled by polarity factors and necessary for proper spindle alignment (reviewed in [78], and asymmetric localization of Bub2-Bfa1. Future work will certainly shed new light onto possible additional similarities in the mechanisms adopted by different organisms to achieve correct spindle positioning.

## Materials and Methods

### Strains and plasmids, media and reagents, genetic manipulations

All strains, except those used for Fig. 7B and 8 (derivatives of S288c) are derivatives of W303 (*ade2–1, trp1–1, leu 2–3,112, his 3–11,15, ura3, ssd1*) and listed in S2 Table. Cells were grown in YEP medium (1% yeast extract, 2% bactopeptone and 50mg/L adenine) supplemented with 2% glucose (YEPD) or 2% galactose (YEPG). Unless otherwise stated, α-factor, nocodazole and benomyl were used at 2, 15 and 12,5 μg/ml respectively. Synchronization experiments with α-factor were performed at 25°C. Bacterial cells were grown in LD broth (1% bactotryptone, 0,5% yeast extract and 0,5% NaCl pH7,25) supplemented with 50 µg/ml ampicillin and 34 μg/ml chloramphenicol.

The *SPC72-BUB2* fusion was generated by triple ligation of a HindIII/XbaI PCR fragment containing the whole ORF of 340 bp of *SPC72* promoter, a XbaI/EcoRI PCR fragment containing the ORF of *BUB2* spanning codons 2–172, and the *LEU2*-based Yiplac128 vector linearized with HindIII and EcoRI. The generated plasmid (pSP275) was linearized with BamHI for integration at the *BUB2* locus, thereby generating a gene fusion under the *SPC72* promoter and containing the whole ORF of *SPC72* fused in frame to the entire ORF of *BUB2*, as well as a truncated *BUB2* gene lacking the last 134 codons. Single integration of the construct at the *BUB2* locus was checked by Southern blot.

The *SPC72-BFA1* fusion was generated by triple ligation of a HindIII/XbaI PCR fragment containing the whole ORF of 340 bp of *SPC72* promoter, a XbaI/BglII PCR fragment containing the entire ORF of *BFA1* starting from the 2^nd^ codon and 330 bp of 3’ UTR, and the *LEU2*-based Yiplac128 vector linearized with HindIII and BamHI. The generated plasmid (pSP371) was linearized with PstI for integration at the *SPC72* locus and single integration of the construct at the *SPC72* locus was checked by Southern blot.

The ORF of *TEM1* and about 1000 bp of promoter region was cloned in Yiplac128 (pSP596). A variant carrying the *TEM1-Q79L* mutation was generated by site-directed mutagenesis (pSP597). The *TEM1*-bearing plasmids have been integrated at the *LEU2* locus by BstXI digestion and single integrations have been checked by Southern blot.

Gene deletions were generated by one-step gene replacement [79]. One-step tagging techniques [80, 81] were used to tag Tem1, Tem1-Q79L, Bfa1, Bub2, Spc72-Bfa1 and Kar9 with multiple HA tags or eGFP.

### Protein expression and purification

*E. coli* BL21 cells carrying pLysE plasmid (Novagen) and *6His-TEM1, 6xHis-TEM1-Q79L, MBP-BFA1, GST-BUB2* and *GST-BUB2-Q132L* expression plasmids were grown in LD broth containing ampicillin and chloramphenicol at 37°C for 3 h, transferred to 14°C for 1 h and induced with 0,1 mM isopropyl-1-thio-β-D-galactopyranoside for 15 h. Cells expressing MBP-Bfa1, 6His-Tem1 and GST-Bub2 fusions were resuspended, respectively, in the following cold lysis buffers: 50 mM Tris-HCl pH7.5, 200 mM NaCl and 2mM DTT supplemented with a cocktail of protease inhibitors (Complete; Boehringer); 50 mM Tris-HCl pH8, 300 mM NaCl, 2mM MgCl_2_ and 10mM imidazole supplemented with a cocktail of protease inhibitors; 50 mM Tris-HCl pH7.5, 200 mM NaCl supplemented with a cocktail of protease inhibitors. Cells were incubated with 1 mg/ml lysozyme in ice for 30 min, placed at 37°C for 5 min and sonicated at 4°C for 10 seconds. The extract was then clarified by centrifugation at 15,000 rpm for 30 min at 4°C. Tem1–6xHis and Tem1-Q79L-6xHis were purified by affinity chromatography with Ni-NTA columns (QUIAGEN). The MBP-Bfa1 fusion protein was purified using Amylose resin (New England Biolabs, Inc.), whereas GST-Bub2 and GST-Bub2-Q132L were purified with glutathione-Sepharose (GE Heathcare). After elution, the fusion proteins were dialyzed against 50 mM Tris-HCl pH7.5, 200 mM NaCl and stored at -80°C. For quantification, purified proteins were analyzed by Comassie staining and by Western blot with anti-GST polyclonal antibodies (Santa Cruz Biotechnology, Inc.), anti-MBP mAb (New England Biolabs, Inc.) and 6xHis mAb (CLONTECH Laboratories, Inc.).

### GTPase assay and dissociation assay

GTPase assay were performed according to [25]. In brief, 240 nM of Tem1–6×His was incubated in 25 μl of loading buffer (20 mM Tris-HCl, pH 7.5, 25 mM NaCl, 5 mM MgCl_2_, and 0.1 mM DTT) containing 0.1 MBq of γ[^32^P]GTP or 0.03 MBq of γ[^35^S]GTP in the absence or presence of 150 nM of MBP-Bfa1 for 10 min at 30°C. The reaction was then put on ice, and 10 μl of reaction were added to 50 μl of reaction buffer (20 mM Tris-HCl, pH 7.5, 2 mM GTP, and 0.6 μg/μl BSA) containing 15 μM of GST-Bub2. The mixture was incubated at 30°C, and for each time point 10 μl of the reaction was diluted in 990 μl of cold washing buffer (20 mM Tris-HCl, pH 7.5, 50 mM NaCl, and 5 mM MgCl_2_). The samples were filtered through nitrocellulose filters, washed with 12 ml of cold washing buffer, and air dried, and the filter-bound radioactivity nucleotide was determined by scintillation counting.

### Immunoprecipitation and western blot analysis

Immunoprecipitations were performed as described in [82]. Bub2-HA3, Bub2-Q132L-HA3, Tem1-HA3 and Tem1-Q79L-HA3 were immunoprecipitated from 1 mg of total extract by a HA-affinity resin (Roche). For Western blot analysis, protein extracts were prepared according to [83]. Proteins transferred to Protran membranes (Schleicher & Schuell) were probed with anti-PK mouse monoclonal antibodies for PK-tagged Bub2, with anti-GFP rat monoclonal antibodies for GFP-tagged Tem1, Bub2 and Bfa1 (Chromotek) and with an anti-HA monoclonal antibody (12CA5). Secondary antibodies were purchased from GE Healthcare, and proteins were detected by an enhanced chemiluminescence system according to the manufacturer.

### Fluorescence microscopy

In situ immunofluorescence was performed according to [84]. Immunostaining of α-tubulin was performed with the YOL34 monoclonal antibody (Serotec) followed by indirect immunofluorescence using rhodamine-conjugated anti–rat antibody (1:100; Pierce Chemical Co.).

Cells expressing GFP and mCherry-tagged proteins were grown in minimum complete medium. Digital images of live cells, cells fixed with 3.7% formaldehyde or cold EtOH were taken with an oil 63X 1,4–1,6 HCX Plan-Apochromat objective (Zeiss) with a Coolsnap HQ2–1 charge device camera (Photometrics) mounted on a ZeissAxioimager Z1/Apotome fluorescence microscope controlled by the MetaMorph imaging system software. Z-stacks of 12 planes at 0.3 μm step size were acquired.

For analysis of actomyosin ring contraction (Fig. 4C) cells were mounted in SD medium on Fluorodishes and filmed at room temperature (~21°C) with a DeltaVision OMX microscope using a 63X 1.4 NA oil immersion objective and the softWoRx software (Applied Precision). Z stacks containing 31 planes were acquired every 1’ with a step size of 0.2 μm and a binning of 1. Z-stacks were deconvolved with Huygens (Scientific Volume Imaging) and max-projected.

For the analyses in Fig. 8, 120s time-lapse microscopy was performed using an Olympus BX51 microscope controlled by the TILLVision software (TILLPhotonics). For the localization of Kar9-YFP and Bub2-GFP, Z-stacks of four layers (step size 0.35 μm) and maximum intensity projections were used. Fluorescence microscopy was performed with a monochromator PolychromIV as light source and a CCD camera (Imago, TillPhotonics).

### Image analysis and processing

Fluorescence intensity measurements of max intensity-projected images were performed using the ImageJ software. The index of Bfa1 symmetric distribution (σ, Fig. 5G) was measured using the following equation: σ _(0<σ<1)_ = *I*_1_/*I*_2_, where *I*_1_ is the fluorescence intensity of the brightest of the two SPBs, and *I*_2_ is the fluorescence intensity of the dimmest.

The position and the orientation of the spindle were measured on max intensity-projected images using ImageJ. The position was determined measuring the distance between the bud neck and the nearest SPB. The orientation was determined measuring the smaller of the two angles that the spindle forms intersecting the polarity axis of the cell.

Adobe Photoshop and ImageJ were used to mount the images and to produce merged color images. No manipulations other than contrast and brightness adjustments were used.

Student’s t-test or chi-square test was used to evaluate statistical significance of differences, depending on whether one (t-test) or more parameters (chi-square test) were compared for each experimental condition.

### Other techniques

Nuclear division was scored with a fluorescence microscope on cells stained with propidium iodide (Sigma Aldrich). Flow cytometric DNA quantification was determined according to [84] on a Becton-Dickinson FACScalibur.

## Supporting Information

S1 Fig The Spc72-Bub2 chimeric protein is SPOC-deficient. The percentage of cells with binucleate cell bodies accompanied or not by SPOC defect was scored after DAPI staining of cycling cells of the indicated strains shifted to 14°C for 16h.
(PDF)

S2 Fig The Spc72-Bfa1 fusion protein suppresses the growth defects of some MEN mutants. **A-E:** Serial dilutions of stationary phase cells with the indicated genotypes were spotted on YPD and incubated at the indicated temperatures.
(PDF)

S3 Fig Increased symmetry of Bfa1 in *TEM1-Q79L* cells does not depend on precocious activation of Cdc15. Logarithmically growing cells with the indicated genotypes were shifted to 37°C for 3 hours and the percentage of cell with symmetric or asymmetric Bfa1-eGFP was scored in anaphase cells stained with DAPI (n≥80).
(PDF)

S1 Table The presence of 9 myc epitopes at the C-terminus of the Spc72-Bub2 chimeric protein causes synthetic sickness to *cdc5–2* and *tem1–3* mutants. Genetic interactions were analysed by crossing the indicated strains followed by tetrad analysis.
(PDF)

S2 Table *S. cerevisiae* strains used in this study. (DOCX)
