# Supplementary material for: Asymmetry of the Budding Yeast Tem1 GTPase at Spindle Poles Is Required for Spindle Positioning But Not for Mitotic Exit
Source: PLoS Genet. 2015 Feb 6;11(2):e1004938. doi: 10.1371/journal.pgen.1004938 (PMC4450052; doi:10.1371/journal.pgen.1004938)
Supplement: S1 Fig — The percentage of cells with binucleate cell bodies accompanied or not by SPOC defect was scored after DAPI staining of cycling cells of the indicated strains shifted to 14°C for 16h. (PDF) [file pgen.1004938.s001.pdf]

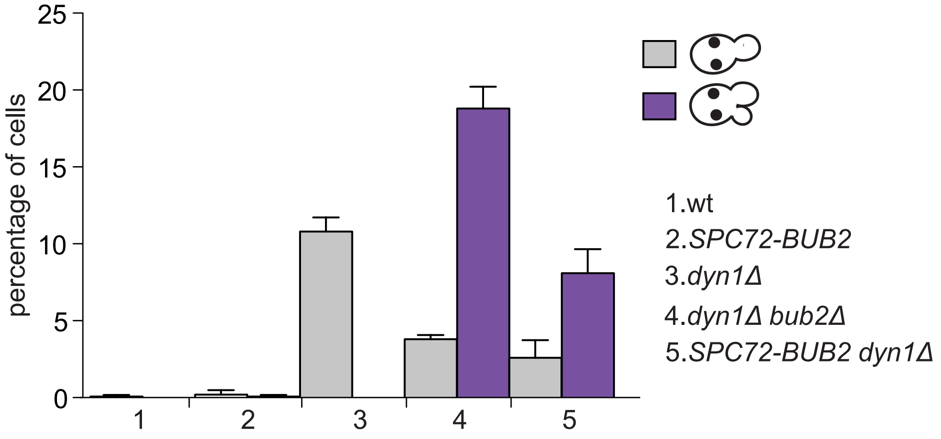

wt
dyn1 Δ
SPC72-BFA1
dyn1Δbub2 Δ
dyn1Δ SPC72-BFA1
dyn1Δbub2Δ SPC72-BFA1
dyn1Δkin4 Δ
dyn1Δkin4Δ SPC72-BFA1
dyn1Δbub2Δkin4Δ SPC72-BFA1
