# Supplementary figures and images for: Asymmetry of the Budding Yeast Tem1 GTPase at Spindle Poles Is Required for Spindle Positioning But Not for Mitotic Exit
Source: PLoS Genet. 2015 Feb 6;11(2):e1004938. doi: 10.1371/journal.pgen.1004938 (PMC4450052; doi:10.1371/journal.pgen.1004938)

**A**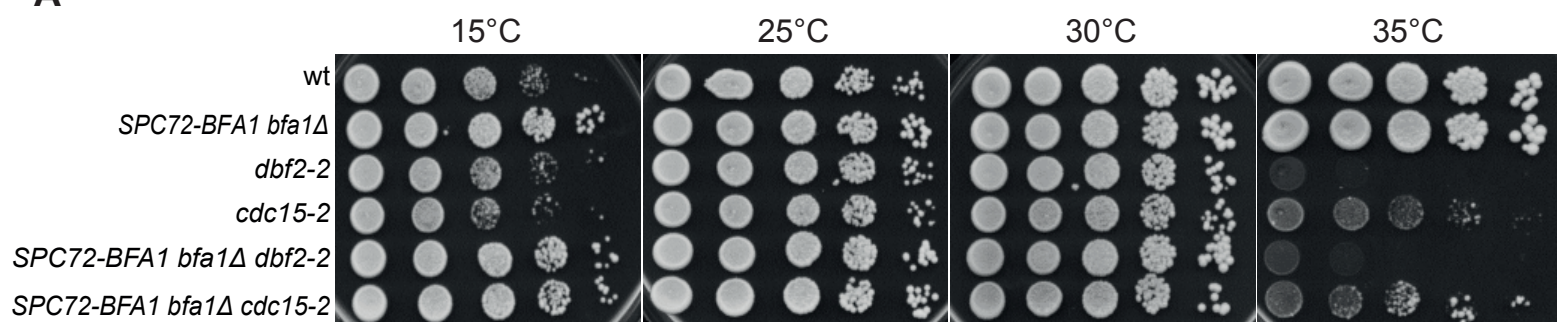**B**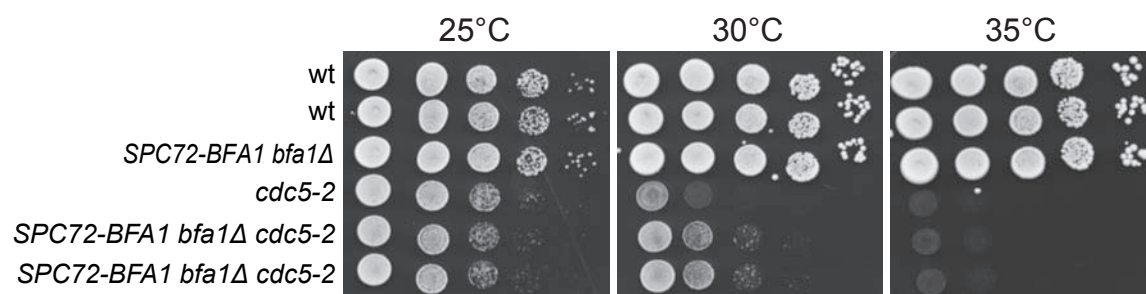**C**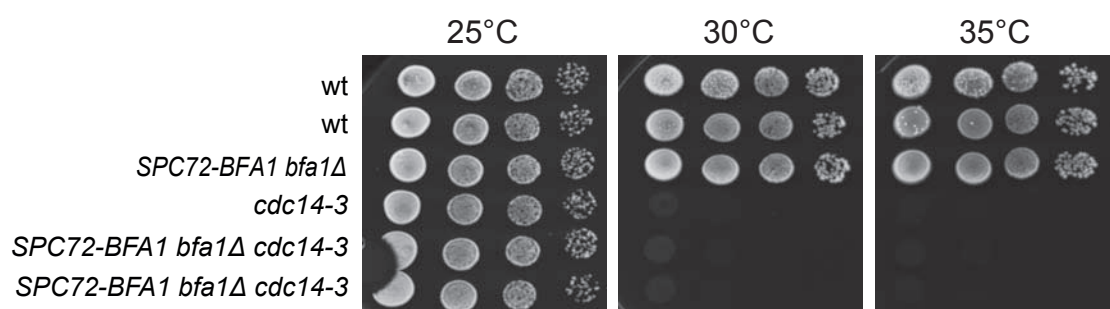**D**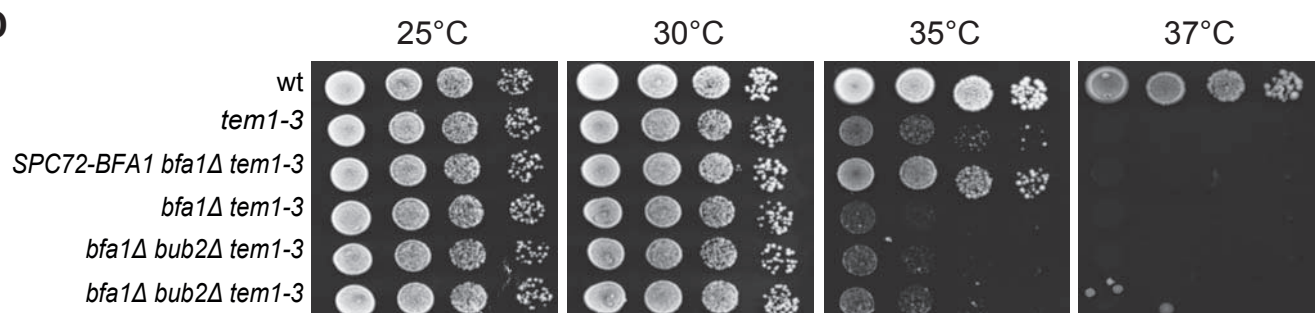**E**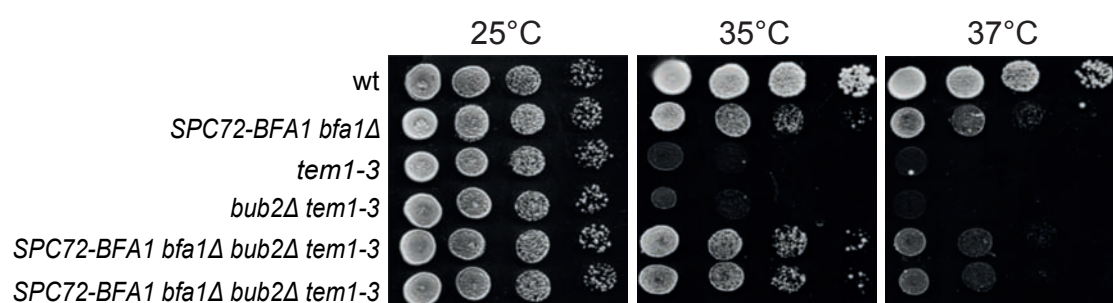

Supplement: S2 Fig — A-E: Serial dilutions of stationary phase cells with the indicated genotypes were spotted on YPD and incubated at the indicated temperatures. (PDF) [file pgen.1004938.s002.pdf]

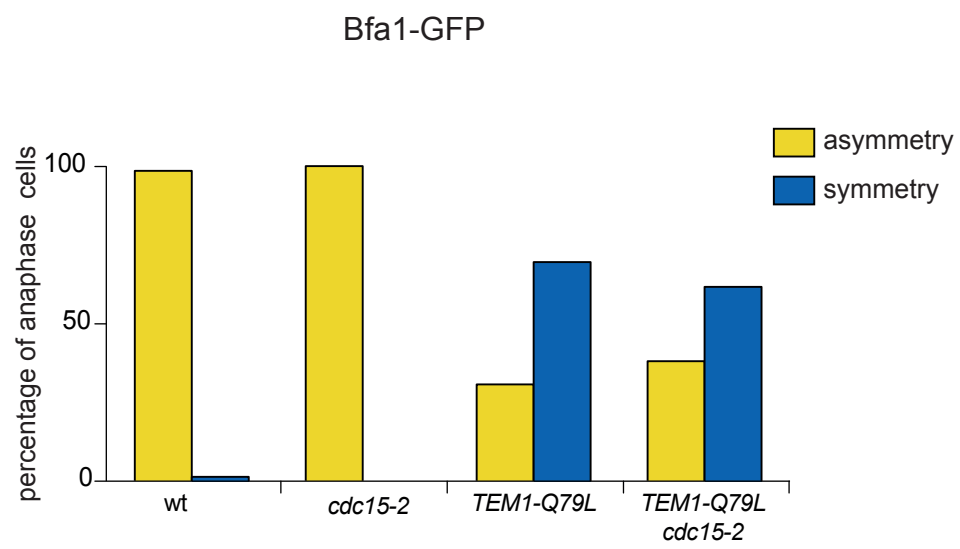

Supplement: S3 Fig — Logarithmically growing cells with the indicated genotypes were shifted to 37°C for 3 hours and the percentage of cell with symmetric or asymmetric Bfa1-eGFP was scored in anaphase cells stained with DAPI (n≥80). (PDF) [file pgen.1004938.s003.pdf]
