# Supplementary material for: Asymmetry of the Budding Yeast Tem1 GTPase at Spindle Poles Is Required for Spindle Positioning But Not for Mitotic Exit
Source: PLoS Genet. 2015 Feb 6;11(2):e1004938. doi: 10.1371/journal.pgen.1004938 (PMC4450052; doi:10.1371/journal.pgen.1004938)
Supplement: S1 Table — Genetic interactions were analysed by crossing the indicated strains followed by tetrad analysis. (PDF) [file pgen.1004938.s004.pdf]

TABLE S1: TETRAD ANALYSIS AFTER CROSSING SEVERAL *BUB2* ALLELES WITH *tem1-3* OR *cdc5-2*

wt x *tem1-3* (26 tetrads)				
	wt	*tem1-3*		
viable	49	47		
inviable	3	5		
expected	52	52		
BUB2-myc9 x *tem1-3* (28 tetrads)				
	wt	*tem1-3*	*BUB2-myc9*	*tem1-3 BUB2-myc9*
viable	21	31	31	2
inviable	0	2	2	19
expected	28	28	28	28
BUB2-HA3 x *tem1-3* (26 tetrads)				
	wt	*tem1-3*	*BUB2-HA3*	*tem1-3 BUB2-HA3*
viable	28	23	24	28
dead	0	1	0	0
expected	26	26	26	26
SPC72-BUB2 x *tem1-3* (24 tetrads)				
	wt	*tem1-3*	*SPC27-BUB2*	*SPC72-BUB2 tem1-3*
viable	22	24	26	21
inviable	0	2	0	1
expected	24	24	24	24
SPC72-BUB2-myc9 x *tem1-3* (27 tetrads)				
	wt	*tem1-3*	*SPC72-BUB2-myc9*	*tem1-3 SPC72-BUB2-myc9*
viable	24	29	29	24*
inviable	0	1	1	0
expected	27	27	27	27
SPC72-BUB2-HA3 x *tem1-3* (22 tetrads)				
	wt	*tem1-3*	*SPC72-BUB2-HA3*	*tem1-3 SPC72-BUB2-HA3*
viable	24	20	19	22
inviable	0	0	1	2
expected	22	22	22	22

* synthetic sickness

wt x *cdc5-2* (27 tetrads)				
	wt	*cdc5-2*		
viable	52	51		
inviable	2	3		
expected	54	54		
BUB2-myc9 x *cdc5-2* (27 tetrads)				
	wt	*cdc5-2*	*BUB2-myc9*	*cdc5-2 BUB2-myc9*
viable	18	34	36	0
inviable	0	2	0	18
expected	27	27	27	27
BUB2-HA3 x *cdc5-2* (14 tetrads)				
	wt	*cdc5-2*	*BUB2-HA3*	*cdc5-2 BUB2-HA3*
viable	11	16	14	11
inviable	0	1	3	0
expected	14	14	14	14
SPC72-BUB2 x *cdc5-2* (19 tetrads)				
	wt	*cdc5-2*	*SPC72-BUB2*	*cdc5-2 SPC72-BUB2*
viable	13	24	25	13
inviable	0	1	0	0
expected	19	19	19	19
SPC72-BUB2-myc9 x *cdc5-2* (28 tetrads)				
	wt	*cdc5-2*	*SPC72-BUB2-myc9*	*cdc5-2 SPC72-BUB2-myc9*
viable	24	31	32	20*
inviable	0	1	0	4
expected	28	28	28	28
SPC72-BUB2-HA3 x *cdc5-2* (21 tetrads)				
	wt	*cdc5-2*	*SPC72-BUB2-HA*	*cdc5-2 SPC72-BUB2-HA*
viable	16	23	24	17
inviable	1	2	1	0
expected	21	21	21	21

* synthetic sickness
