# Supplementary material for: Asymmetry of the Budding Yeast Tem1 GTPase at Spindle Poles Is Required for Spindle Positioning But Not for Mitotic Exit
Source: PLoS Genet. 2015 Feb 6;11(2):e1004938. doi: 10.1371/journal.pgen.1004938 (PMC4450052; doi:10.1371/journal.pgen.1004938)
Supplement: S2 Table — (DOCX) [file pgen.1004938.s005.docx]

**Table S2. List of *Saccharomyces cerevisiae* strains used in this study**

| **S288C background** | |
| --- | --- |
| **Name** | **Relevant genotype** |
| yYB3160 | *MATalpha, bub2::KAN KAR9-YFP:NatNT2, ura3::CFP-TUB1:URA3* |
| yYB3272 | *MATalpha, BFA1-yeGFP:HIS3MX6, ura3::CFP-TUB1:URA3* |
| yYB3302 | *MATalpha, BUB2-R85A::URA3, KAR9-YFP:NatNT2, ura3::CFP-TUB1:URA3* |
| yYB3300 | *MATa, BUB2-myc9::TRP1, KAR9-YFP:NatNT2, ura3::CFP-TUB1:URA3* |
| yYB3303 | *MATalpha, BUB2-Q132L::URA3, KAR9-YFP:NatNT2, ura3::CFP-TUB1:URA3* |
| yYB3305 | *MATa, BUB2-Q132L::URA3, BFA1-yeGFP:HIS3MX6, ura3::CFP-TUB1:URA3* |
| yYB3306 | *MATa, BUB2-R85A::URA3, BFA1-yeGFP:HIS3MX6, ura3::CFP-TUB1:URA3* |
| yYB3338 | *MATa, BUB2-myc9::TRP1 BFA1-yeGFP:HIS3MX6, ura3::CFP-TUB1:URA3* |
| yYB3946 | *MATa, bfa1::KAN KAR9-YFP:NatNT2, ura3::CFP-TUB1:URA3* |
| yYB5569 | *MATa KAR9-YFP:NatNT2, ura3::CFP-TUB1:URA3* |
| yYB7445 | *MATa, KAR9-YFP:NatNT2, SPC42-mCherry:KanMX4, ura3::CFP-TUB1:URA3* |
| yYB10296 | *MATa, BUB2-yeGFP:HIS3MX6, SPC42-mCherry:KanMX4, ura3::CFP-TUB1:URA3* |
| yYB11429 | *MATa, BUB2-yeGFP:HIS3MX6, SPC42-mCherry:KanMX4, ura3::CFP-TUB1:URA3, dyn1::hphNT1* |
| **W303 background** | |
| **Name** | **Relevant genotype** (Plasmids are indicated in brackets) |
| ySP52 | *MATalpha, cdc15-2* |
| ySP285 | *MATalpha, cdc14-3* |
| ySP311 | *MATalpha, dbf2-2* |
| ySP325 | *MATalpha, cdc5-2::URA3* |
| ySP1243 | *MATa, bfa1::TRP1* |
| ySP1959 | *MATa, BFA1-HA6::HIS3* |
| ySP1961 | *MATa, BUB2-HA3::KlTRP1* |
| ySP2035 | *MATalpha, BFA1-HA6::HIS3* |
| ySP3138 | *MATa, bub2::HIS3* |
| ySP3197 | *MATalpha, MYO1-GFP::KanMx4* |
| ySP3417 | *MATa, tem1::URA3, [YCplac111-tem1-3]* |
| ySP3641 | *MATalpha, TEM1-HA3::KlURA3* |
| ySP3673 | *MATalpha, BFA1-GFP::URA3, bfa1::TRP1* |
| ySP3773 | *MATa, BFA1-GFP::URA3, bfa1::TRP1, TEM1-HA3::KlURA3* |
| ySP3895 | *MATa, slk19::HIS3* |
| ySP4091 | *MATalpha, tem1::URA3, [pRS315-TEM1-GFP-KanMX4], BUB2-HA3::KlTRP1* |
| ySP4165 | *MATalpha, tem1::URA3, [pRS315-TEM1-GFP-KanMX4]* |
| ySP4657 | *MATa, SPC72-BUB2::BUB2::LEU2* |
| ySP5581 | *MATalpha, SPC72::SPC72-BFA1::LEU2* |
| ySP5706 | *MATalpha, SPC72::SPC72-BFA1::LEU2, bfa1::TRP1, tem1::URA3, [YCplac111-tem1-3]* |
| ySP5816 | *MATa, bfa1::TRP1, tem1::URA3, [YCplac111-tem1-3]* |
| ySP5817 | *MATalpha, bfa1::TRP1,tem1::URA3, [YCplac111-tem1-3]* |
| ySP5836 | *MATalpha, SPC72::SPC72-BFA1::LEU2, bfa1::TRP1, tem1::URA3, [YCplac111-tem1-3], bub2::HIS3* |
| ySP5837 | *MATa, SPC72::SPC72-BFA1::LEU2, bfa1::TRP1, tem1::URA3, [YCplac111-tem1-3], bub2::HIS3* |
| ySP6270 | *MATa, kar9::KanMX4* |
| ySP6292 | *MATa, dyn1::KanMX4* |
| ySP6312 | *MATa, bub2::bub2-Q132L::URA3* |
| ySP6432 | *MATalpha, bub2::bub2-Q132L-HA3::TRP1::URA3* |
| ySP6435 | *MATa, bub2::bub2-Q132L::URA3, BFA1-HA6::HIS3* |
| ySP6495 | *MATa, dyn1::KanMX4, bub2::bub2-Q132L::URA3* |
| ySP6508 | *MATalpha, kar9::KanMX4, bub2::bub2-Q132L::URA3* |
| ySP6552 | *MATalpha, dyn1::KanMX4* |
| ySP6609 | *MATa, bub2::Q132L-HA3::TRP1::URA3, tem1::URA3, [pRS315-TEM1-GFP-KanMX4]* |
| ySP6678 | *MATa, bub2::HIS3, kar9::KanMX4* |
| ySP6680 | *MATa, bub2::HIS3, dyn1::KanMX4* |
| ySP6744 | *MATalpha, cdc5-2::URA3, SPC72::SPC72-BFA1::LEU2, bfa1::TRP1* |
| ySP6745 | *MATa, cdc5-2::URA3, SPC72::SPC72-BFA1::LEU2, bfa1::TRP1* |
| ySP6775 | *MATalpha, bub2::bub2-Q132L-HA3::TRP1::URA3, BFA1-GFP::URA3, bfa1::TRP1* |
| ySP6798 | *MATa, BFA1-GFP::URA3, bfa1::TRP1, BUB2-HA3::KlTRP1* |
| ySP7214 | *MATalpha, tem1::URA3, LEU2::TEM1* |
| ySP7217 | *MATa, tem1::URA3, LEU2::TEM1-Q79L* |
| ySP7218 | *MATalpha, tem1::URA3, LEU2::TEM1-HA3* |
| ySP7221 | *MATa, tem1::URA3, LEU2::TEM1-Q79L-HA3* |
| ySP7270 | *MATalpha, BFA1-GFP::URA3, bfa1::TRP1, tem1::URA3, LEU2::TEM1-Q79L-HA3* |
| ySP7277 | *MATa, tem1::URA3, LEU2::TEM1-HA3, BFA1-GFP::URA3, bfa1::TRP1* |
| ySP7285 | *MATa, BUB2-3PK::K.l.HIS3* |
| ySP7286 | *MATa, TEM1-HA3::K.l.URA3, BUB2-3PK::KlHIS3* |
| ySP7287 | *MATa, tem1::URA3, LEU2::TEM1-Q79L-HA3, BUB2-3PK::KlHIS3* |
| ySP7321 | *MATa, BUB2-3PK::K.l.HIS3, tem1::URA3, LEU2::TEM1-HA3* |
| ySP7633 | *MATalpha, dyn1::KanMX4, tem1::URA3, LEU2::TEM1-Q79L* |
| ySP7790 | *MATa, GAL-ESP1::TRP1* |
| ySP7792 | *MATa, bns1::KanMX4, spo12::HIS3* |
| ySP8713 | *MATa, mCherry-TUB1::URA3, tem1::URA3, LEU2::TEM1-Q79L, BFA1-eGFP::KanMX4* |
| ySP9164 | *MATa, GAL-KIN4::TRP1* |
| ySP9448 | *MATa, BFA1::BFA1-eGFP::KanMX4, mCherry-TUB1::URA3.* |
| ySP9534 | *MATa, BFA1::BFA1-eGFP::KanMX4, mCherry-TUB1::URA3, kin4::KanMX4.* |
| ySP9545 | *MATa, tem1::URA3, LEU2::TEM1-Q79L, BFA1::BFA1-eGFP::KanMX4, kin4::KanMX4, mCherry-TUB1::URA3* |
| ySP9623 | *MATa, BFA1::BFA1-eGFP::KanMX4, mCherry-TUB1::URA3, cdc15-2* |
| ySP9665 | *MATalpha, tem1::URA3, LEU2::TEM1-Q79L-HA3, CDC15-GFP::KanMX4, mCherry-TUB1::URA3* |
| ySP9666 | *MATalpha, CDC15-GFP::KanMX4, tem1::URA3, LEU2::TEM1-HA3, mCherry-TUB1::URA3* |
| ySP9669 | *MATa, mCherry-TUB1::URA3, tem1::URA3, LEU2::TEM1-Q79L, BFA1-eGFP::KanMX4, cdc15-2* |
| ySP9691 | *MATa, SPC72::SPC72-BFA1::LEU2, bfa1::TRP1* |
| ySP9697 | *MATa, tem1::URA3, LEU2::TEM1-Q79L-HA3, CDC15-GFP::KanMX4, kin4::KanMX4, mCherry-TUB1::URA3* |
| ySP9702 | *MATalpha, CDC15-GFP::KanMX4, tem1::URA3, LEU2::TEM1-HA3, mCherry-TUB1::URA3, kin4::KanMX4* |
| ySP9736 | *MATalpha, SPC72::SPC72-BFA1::LEU2, bfa1::TRP1, cdc15-2* |
| ySP9738 | *MATalpha, SPC72::SPC72-BFA1::LEU2, bfa1::TRP1, dbf2-2* |
| ySP9790 | *MATa, SPC72::SPC72-BFA1::LEU2, bfa1::TRP1, dyn1::KanMX4* |
| ySP9793 | *MATa, SPC72::SPC72-BFA1::LEU2, bfa1::TRP1, clb2::LEU2* |
| ySP9797 | *MATa, LEU2::TEM1-Q79L* |
| ySP9798 | *MATalpha, LEU2::TEM1-Q79L* |
| ySP9825 | *MATalpha, tem1::URA3, LEU2::tem1Q79L, MYO1-GFP::KanMx4* |
| ySP9829 | *MATa, mCherry-TUB1::URA3, mob1::HIS3, GFP-MOB1::URA3* |
| ySP9830 | *MATa, LEU2::TEM1-Q79L, mCherry-TUB1::URA3, mob1::HIS3, GFP-MOB1::URA3* |
| ySP9831 | *MATalpha, SPC72::SPC72-BFA1::LEU2, bfa1::TRP1, dyn1::KanMX4, kin4::KanMX4* |
| ySP9833 | *MATalpha, LEU2::TEM1-Q79L, clb2::LEU2* |
| ySP9859 | *MATalpha, SPC72::SPC72-BFA1::LEU2, bfa1::TRP1, cdc14-3* |
| ySP9860 | *MATa, SPC72::SPC72-BFA1::LEU2, bfa1::TRP1, cdc14-3* |
| ySP9868 | *MATa, dyn1::KanMX4, kin4::KanMX4* |
| ySP9870 | *MATa, bns1::KanMX4, spo12::HIS3, SPC72::SPC72-BFA1::LEU2, bfa1::TRP1* |
| ySP9890 | *MATalpha, GAL-KIN4::TRP1, SPC72::SPC72-BFA1::LEU2, bfa1::TRP1* |
| ySP9913 | *MATa, cdc55::TRP1* |
| ySP9915 | *MATa, bns1::KanMX4, spo12::HIS3, SPC72::SPC72-BFA1::LEU2, bfa1::TRP1, dyn1::KanMX4* |
| ySP9916 | *MATalpha, bns1::KanMX4, spo12::HIS3, dyn1::KanMX4* |
| ySP9935 | *MATa, LEU2::tem1-Q79L, clb2::LEU2* |
| ySP9938 | *MATa, cdc55::TRP1, SPC72::SPC72-BFA1::LEU2, bfa1::TRP1* |
| ySP9977 | *MATa, KAR9-eGFP::KanMX4, SPC42-mCherry::NatN2* |
| ySP9998 | *MATalpha, SPC72-BUB2::BUB2::LEU2* |
| ySP10042 | *MATa, LEU2::TEM1-Q79L, GAL-KIN4::TRP1* |
| ySP10046 | *MATa, SPC72::SPC72-BFA1::LEU2, bfa1::TRP1, TEM1-eGFP::KanMX4, mCherry-TUB1::URA3* |
| ySP10047 | *MATalpha, CDC15-GFP::KanMX4, SPC72::SPC72-BFA1::LEU2, bfa1::TRP1, mCherry-TUB1::URA3* |
| ySP10064 | *MATa, TEM1-eGFP::KanMX4, mCherry-TUB1::URA3* |
| ySP10065 | *MATalpha, TEM1-eGFP::KanMX4, mCherry-TUB1::URA3* |
| ySP10066 | *MATa, CDC15-GFP::KanMX4, mCherry-TUB1::URA3* |
| ySP10069 | *MATa, SPC72::SPC72-BFA1-eGFP::KanMX4::LEU2, bfa1::TRP, SPC42-mCherry::NatN2* |
| ySP10157 | *MATa, clb2::LEU2* |
| ySP10162 | *MATalpha, SPC72-BUB2::BUB2::LEU2, dyn1::KanMX4* |
| ySP10268 | *MATa, KAR9-eGFP::KanMX4, SPC42-mCherry::NatN2, tem1::URA3, LEU2::TEM1-Q79L* |
| ySP10289 | *MATa, SPC72-BUB2::BUB2::LEU2, KAR9-eGFP::KanMX4, SPC42-mCherry::NatN2* |
| ySP10290 | *MATa, SPC72::SPC72-BFA1::LEU2, bfa1::TRP1, KAR9-eGFP::KanMX4, SPC42-mCherry::NatN2* |
| ySP10392 | *MATa, GAL-KIN4::TRP1, SPC72::SPC72-BFA1::LEU2* |
| ySP10399 | *MATalpha, GAL-KIN4::TRP1, bfa1::TRP1* |
| ySP10447 | *MATa, SPC72::SPC72-BFA1::LEU2, tem1::URA3, [YCplac111-tem1-3]* |
| ySP10472 | *MATalpha, tem1-3* |
| ySP10560 | *MATa, mob1-77::URA3* |
| ySP10594 | *MATa, SPC72-BUB2::BUB2::LEU2, cdc55::TRP1* |
| ySP10622 | *MATa, mob1-77::URA3, SPC72::SPC72-BFA1::LEU2, bfa1::TRP1* |
| ySP11224 | *MATa, bfa1::TRP1, tem1::URA3, [YCplac111-tem1-3], bub2::HIS3* |
| ySP11225 | *MATa, bfa1::TRP1, tem1::URA3, [YCplac111-tem1-3], bub2::HIS3* |
| ySP11453 | *MATa, CDC15-GFP::KanMX4, mCherry-TUB1::URA3, bub2::bub2-Q132L::URA3* |
| ySP11452 | *MATa, CDC15-GFP::KanMX4, mCherry-TUB1::URA3, bub2::HIS3* |
| ySP11456 | *MATa, SPC72-BUB2::BUB2::LEU2, GAL-ESP1::TRP1* |
| ySP11492 | *MATa, SPC72::SPC72-BFA1::LEU2, bfa1::TRP1, GAL-ESP1::TRP1* |
| ySP11511 | *MATa, SPC72::SPC72-BFA1::LEU2, bfa1::TRP1, slk19::HIS3* |
| ySP11513 | *MATa, SPC72::SPC72-BFA1::LEU2, bfa1::TRP1, dyn1::KanMx4, slk19::HIS3* |
| ySP11515 | *MATa, dyn1::KanMx4, slk19::HIS3* |
| ySP11534 | *MATa, TEM1-eGFP::KanMX4, mCherry-TUB1::URA3, bub2::bub2-Q132L::URA3* |
| ySP11536 | *MATa, BFA1-eGFP::KanMX4, mCherry-TUB1::URA3, bub2::bub2Q132L::URA3* |
| ySP11583 | *MATa, tem1::HPHMx [pRS315-TEM1-GFP-kanM46], mCherry-TUB1::URA3* |
| ySP11585 | *MATa, tem1::HPHMx [pRS315-TEM1-Q79L-GFP-kanMX4], mCherry-TUB1::URA3* |
| ySP11619 | *MATa, BUB2-eGFP::KanMx4, mCherry-TUB1::URA3* |
| ySP11621 | *MATa, bub2-Q132L-eGFP::KanMx4, mCherry-TUB1::URA3* |
| ySP11623 | *MATalpha, BFA1-GFP::URA3, bfa1::TRP1, BUB2-eGFP::KanMX4* |
| ySP11624 | *MATalpha, BFA1-GFP::URA3, bfa1::TRP1, tem1::URA3, LEU2::tem1Q79L-HA3, BUB2-eGFP::KanMX4* |
| ySP11625 | *MATa, tem1::URA3, LEU2::TEM1-HA3, BFA1-GFP::URA3, bfa1::TRP1, BUB2-eGFP::KanMX4* |
| ySP11673 | *MATa, tem1::HPHMx [pRS315-TEM1-GFP-kanM46], mCherry-TUB1::URA3, bfa1::TRP1* |
| ySP11675 | *MATa, tem1::HPHMx [pRS315-TEM1-Q79L-GFP-kanM46], mCherry-TUB1::URA3, bfa1::TRP1* |
|  |  |
